# Structural and functional basis of inositol hexaphosphate stimulation of NHEJ through stabilization of Ku-XLF interaction

**DOI:** 10.1093/nar/gkad863

**Published:** 2023-10-23

**Authors:** Antonia Kefala Stavridi, Amandine Gontier, Vincent Morin, Philippe Frit, Virginie Ropars, Nadia Barboule, Carine Racca, Sagun Jonchhe, Michael J Morten, Jessica Andreani, Alexey Rak, Pierre Legrand, Alexa Bourand-Plantefol, Steven W Hardwick, Dimitri Y Chirgadze, Paul Davey, Taiana Maia De Oliveira, Eli Rothenberg, Sebastien Britton, Patrick Calsou, Tom L Blundell, Paloma F Varela, Amanda K Chaplin, Jean-Baptiste Charbonnier

**Affiliations:** Heartand Lung Research Institute, University of Cambridge, Biomedical Campus, Papworth Road, Trumpington, Cambridge CB2 0BB, UK; Institute for Integrative Biology of the Cell (I2BC), Institute Joliot, CEA, CNRS, Univ.Paris-Sud, Université Paris-Saclay, 91198, Gif-sur-Yvette cedex, France; Institute for Integrative Biology of the Cell (I2BC), Institute Joliot, CEA, CNRS, Univ.Paris-Sud, Université Paris-Saclay, 91198, Gif-sur-Yvette cedex, France; Institut de Pharmacologie et Biologie Structurale (IPBS), Université de Toulouse, CNRS, Université Toulouse III - Paul Sabatier (UT3), Toulouse, France; Equipe Labellisée Ligue Contre le Cancer 2018, Toulouse, France; Institute for Integrative Biology of the Cell (I2BC), Institute Joliot, CEA, CNRS, Univ.Paris-Sud, Université Paris-Saclay, 91198, Gif-sur-Yvette cedex, France; Institut de Pharmacologie et Biologie Structurale (IPBS), Université de Toulouse, CNRS, Université Toulouse III - Paul Sabatier (UT3), Toulouse, France; Equipe Labellisée Ligue Contre le Cancer 2018, Toulouse, France; Institut de Pharmacologie et Biologie Structurale (IPBS), Université de Toulouse, CNRS, Université Toulouse III - Paul Sabatier (UT3), Toulouse, France; Equipe Labellisée Ligue Contre le Cancer 2018, Toulouse, France; NYU Langone Medical Center, 450 East 29th Street, NY, NY, USA York University, USA; NYU Langone Medical Center, 450 East 29th Street, NY, NY, USA York University, USA; Institute for Integrative Biology of the Cell (I2BC), Institute Joliot, CEA, CNRS, Univ.Paris-Sud, Université Paris-Saclay, 91198, Gif-sur-Yvette cedex, France; Structure-Design-Informatics, Sanofi R&D, Vitry sur Seine, France; Synchrotron SOLEIL, L’Orme des Merisiers, Saint-Aubin, Gif-sur-Yvette, France; Institute for Integrative Biology of the Cell (I2BC), Institute Joliot, CEA, CNRS, Univ.Paris-Sud, Université Paris-Saclay, 91198, Gif-sur-Yvette cedex, France; Cryo-EM Facility, Department of Biochemistry, University of Cambridge, Cambridge CB2 1GA, UK; Cryo-EM Facility, Department of Biochemistry, University of Cambridge, Cambridge CB2 1GA, UK; Oncology, R&D, AstraZeneca, Cambridge, UK; Mechanistic and Structural Biology, Discovery Sciences, R&D, AstraZeneca, Cambridge, UK; NYU Langone Medical Center, 450 East 29th Street, NY, NY, USA York University, USA; Institut de Pharmacologie et Biologie Structurale (IPBS), Université de Toulouse, CNRS, Université Toulouse III - Paul Sabatier (UT3), Toulouse, France; Equipe Labellisée Ligue Contre le Cancer 2018, Toulouse, France; Institut de Pharmacologie et Biologie Structurale (IPBS), Université de Toulouse, CNRS, Université Toulouse III - Paul Sabatier (UT3), Toulouse, France; Equipe Labellisée Ligue Contre le Cancer 2018, Toulouse, France; Heartand Lung Research Institute, University of Cambridge, Biomedical Campus, Papworth Road, Trumpington, Cambridge CB2 0BB, UK; Institute for Integrative Biology of the Cell (I2BC), Institute Joliot, CEA, CNRS, Univ.Paris-Sud, Université Paris-Saclay, 91198, Gif-sur-Yvette cedex, France; Leicester Institute for Structural and Chemical Biology, Department of Molecular and Cell Biology, University of Leicester, Leicester, UK; Institute for Integrative Biology of the Cell (I2BC), Institute Joliot, CEA, CNRS, Univ.Paris-Sud, Université Paris-Saclay, 91198, Gif-sur-Yvette cedex, France

## Abstract

The classical Non-Homologous End Joining (c-NHEJ) pathway is the predominant process in mammals for repairing endogenous, accidental or programmed DNA Double-Strand Breaks. c-NHEJ is regulated by several accessory factors, post-translational modifications, endogenous chemical agents and metabolites. The metabolite inositol-hexaphosphate (IP6) stimulates c-NHEJ by interacting with the Ku70–Ku80 heterodimer (Ku). We report cryo-EM structures of apo- and DNA-bound Ku in complex with IP6, at 3.5 Å and 2.74 Å resolutions respectively, and an X-ray crystallography structure of a Ku in complex with DNA and IP6 at 3.7 Å. The Ku-IP6 interaction is mediated predominantly via salt bridges at the interface of the Ku70 and Ku80 subunits. This interaction is distant from the DNA, DNA-PKcs, APLF and PAXX binding sites and in close proximity to XLF binding site. Biophysical experiments show that IP6 binding increases the thermal stability of Ku by 2°C in a DNA-dependent manner, stabilizes Ku on DNA and enhances XLF affinity for Ku. In cells, selected mutagenesis of the IP6 binding pocket reduces both Ku accrual at damaged sites and XLF enrolment in the NHEJ complex, which translate into a lower end-joining efficiency. Thus, this study defines the molecular bases of the IP6 metabolite stimulatory effect on the c-NHEJ repair activity.

## Graphical Abstract

**Graphical Abstract:**
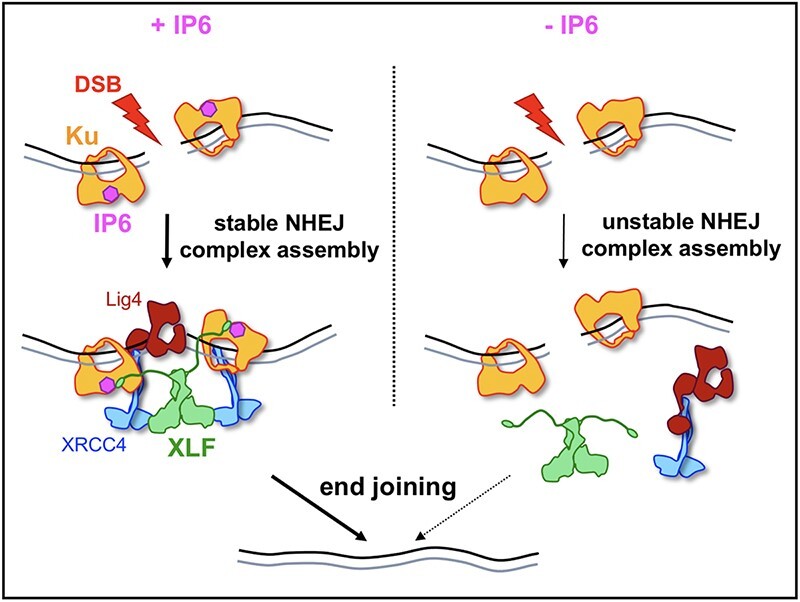

## Introduction

**Figure 1.**
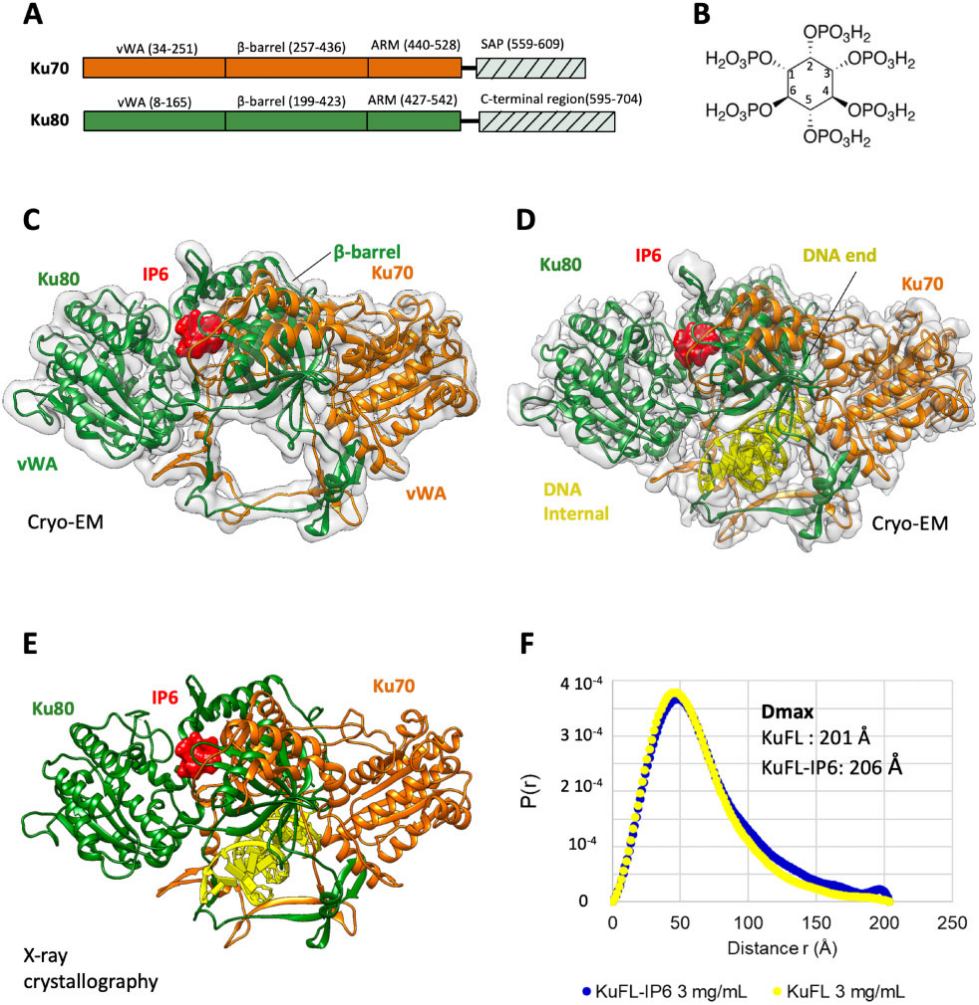
Cryo-EM and crystal structures of the human Ku heterodimers bound to inositol hexaphosphate. (**A**) Domains of Ku70 and Ku80. The C-terminal regions (dash area) are not visible in the cryo-EM structures and are deleted for crystallization of the Ku_ΔC_ construct. The boundaries of the domains are indicated. (**B**) Structure of the inositol hexaphosphate (IP6) with the position and orientation of the phosphates. (C, D) Cryo-EM structure of (**C**) full-length Ku alone and (**D**) Ku bound to a 15 bp DNA with a 15mer 5′ overhang at 3.5 and 2.74 Å resolution, respectively. IP6 co-purified with Ku expressed in Sf9 insect cells. Ku70 is shown in orange, Ku80 in forest green, DNA in yellow and IP6 as a red surface. (**E**) Crystal structure at 3.7 Å resolution of Ku_ΔC_ with a 21–34 bp hairpin DNA (hDNA) and IP6. (**F**) Distance distribution, P(r), from SAXS analysis of full-length Ku with or without IP6.

The heterodimer Ku70–Ku80 (Ku) is a central factor of the classical Non-Homologous End-Joining (c-NHEJ) pathway, the predominant DNA Double-Strand Break (DSB) repair pathway in multicellular organisms (1). It recognizes DSB ends through its ring-shaped structure in a sequence-independent manner. Ku has a nanomolar affinity for DNA ends and is present in abundance within the nucleus (∼500 000 Ku/cell) (2,3). Beyond the damage recognition function, Ku acts as the main hub in the c-NHEJ pathway (4). With the DNA-dependent protein kinase catalytic subunit (DNA-PKcs), it forms the DNA-PK holoenzyme that maintains the two DNA ends in close proximity by DNA ends synapsis and phosphorylates several nearby proteins including itself (5,6). Upon binding to the broken DNA ends, Ku subsequently recruits most of the c-NHEJ factors, including enzymes responsible for end processing (nucleases, kinases and phosphatases), for nucleotide addition (pol X DNA polymerases) and for ligation (the XRCC4/Lig4 complex together with XLF and PAXX co-factors) (4,7). Important advances on molecular understanding of the c-NHEJ process have been gained recently thanks to single-molecule approaches and structural studies (reviewed in (8,9)). The Ku heterodimer is composed of Ku70 and Ku80 proteins, which have a similarly arrangement of vWA, β-barrel and ARM domains, but differ at the C-terminus where Ku70 encodes a SAP domain and Ku80 a globular domain that is stabilised upon interaction with DNA-PK (Figure 1A).

In eukaryotes, inositol phosphates (IPs) regulate several cellular processes including cohesin dynamics, RNA-editing and RNA-export, nuclear transport, post-translational modification and HIV-1 virus assembly and maturation (10–12). IPs are constituted by an inositol functional group (a six carbon cyclic alcohol) and phosphate groups that occupy axial or equatorial positions (Figure 1B for IP6 structure). IPs form a wide array of stereochemically distinct signalling molecules with functions in many cellular pathways from yeast to mammals (11). IP4, IP5 and IP6 are generated from the second messenger inositol 1,4,5-trisphosphate (IP3) by a family of inositol phosphate kinases. IP6 has been previously characterized as a structural or as an enzymatic cofactor. For example, in the RNA editing enzyme adenosine deaminase (ADAR2), IP6 occupies a buried position and contributes to the protein fold (13). An IP6 molecule was also unexpectedly found near the centre of a small F-box protein (TIR1) in close vicinity to the binding site of the plant hormone auxin, suggesting a potential role of IP6 in plant hormone signalling pathways (14).

Two decades ago, IP6 was identified as a regulator of c-NHEJ (15). Using human whole-cell extracts, IP6 was shown to stimulate c-NHEJ through its interaction with DNA-PK. Its removal through phosphocellulose fractionation reduced end-joining efficiency *in vitro*. Subsequent studies showed that IP6 specifically interacts with Ku (16,17). Molecular modelling and mutations of basic residues in Ku70 and Ku80 then allowed proposing a first broad mapping of the IP6 binding site (18). Despite important results from the studies mentioned above, the molecular basis of the role of IP6 on Ku function is poorly understood. Recently, Liu's *et al.* reported a cryoEM structure of DNA-PK bound to Artemis and identified in Ku heterodimer a density that was attributed to IP6 molecule (19).

Here, we present cryo-EM and X-ray crystallography structures of Ku with or without DNA in complex with the IP6 molecule. We characterize the binding site of the IP6 at the interface between the Ku70 and Ku80 subunits. IP6 binds a solvent accessible pocket delineated by basic residues. The pocket is located away from the DNA, DNA-PKcs, ALPF and PAXX sites but is close to the binding site of the Ku-Binding Motif (KBM) of XLF. We use complementary biophysical approaches (calorimetry, thermal denaturation, switchSENSE) and single molecule colocalization to characterize the consequences of IP6 binding to Ku on Ku interactions with DNA and with XLF. Finally, we evaluate the impacts of Ku mutations in the IP6 site on the recruitment of c-NHEJ factors at DSB sites induced by laser micro-irradiation or by a clastogenic molecule and on end-joining efficiency in cells with a new dedicated cellular assay. This study provides an in-depth description of IP6 binding to Ku and a combined biophysical and cellular characterization of its impact on c-NHEJ.

## Materials and methods

### Expression and purification of Ku70–Ku80 (Ku)

Purification of Ku full-length (Ku_FL_) for cryo-EM experiments was carried out as described previously (5). Expression and purification of Ku_FL_ for biophysical experiments was carried out as described previously (20). The full-length human Ku heterodimer subunits (Ku70 1–609, Ku80 1–732) were cloned into a Multibac vector (21). The construct has a 10× His-tag and a TEV site on the N-terminus of Ku80 and no-tag on Ku70. Protein production was initiated in Sf21 cells by infection with the baculovirus stock at MOI of 5 × 10^−3^ and cells were collected 5–6 days after the infection (3–4 days after the proliferation arrest). Cells were sonicated and the supernatant was incubated with Benzonase (300 units for 30 min at 4°C). The Ku heterodimer was purified on a NiNTA-Agarose affinity column (Protino, Macherey Nagel) with a 1 M NaCl wash step to remove DNA excess. The eluted Ku was then bound onto an anion exchange column (Resource Q, Cytiva) equilibrated in 20 mM Tris pH 8.0, 50 mM NaCl, 50 mM KCl, 10 mM β-mercaptoethanol and eluted with a salt gradient. The final yield was about 20 mg of purified heterodimer per litre of culture. Ku_ΔC_ constructs, heterodimer deleted of its C-terminal region (Ku70 1–544, Ku80 1–551) for X-ray crystallography, Ku mutants, Ku-2E (Ku with mutation in K357E on Ku70 and K481E on Ku80) and Ku-4E (Ku with mutations in H359E and H360E on Ku70 and K413E and H414E on Ku80) were cloned in the same vector and produced following the same protocol as Ku_FL_ expressed in Sf21 insect cells.

### Cryo-EM sample preparation, data acquisition, image processing and structures refinement

Apo-Ku or in complex with a 15 bp DNA with a 15mer 5′ overhang (see oligonucleotides in Supplemental Information) at a 1:1.2 ratio, were prepared at final concentrations of 3 mg/mL and 1.7 mg/mL respectively. Prior to plunge-freezing, the Ku-DNA sample was incubated with Methyl-PEG4-NHS-Ester (ThermoFisher) to a final concentration of 2 mM (25°C, 2 h) and the reaction was quenched with 0.1 v/v of 1 M Tris–HCl, pH 8.0. Aliquots of 2.5 μL were then placed on glow-discharged (60 sec at current of 25 mA in PELCO Easiglow (Ted Pella, Inc)) holey carbon grids (Quantifoil Cu R1.2/1.3, 300 mesh) and plunge-frozen in liquid ethane using a FEI Vitrobot Mark IV system (Thermo Fisher Scientific) (4°C, 95% humidity). Cryo-EM data for the Ku70–Ku80-IP6 complex were collected in the Department of Biochemistry, University of Cambridge on a Titan Krios and for the Ku70–Ku80–DNA–IP6 in the Department of Material Sciences, University of Cambridge. Details on the collection parameters can be found in Table S1. 13 140 movies for Ku-IP6 and 6 924 movies for Ku-IP6-DNA were collected in accurate hole centring mode using EPU software (Thermo Fisher Scientific). 485 147 particles for the Ku-IP6 map and 2 143 882 for the Ku-DNA-IP6 map were picked by the BoxNet2Mask neural network model in Warp (22), where CTF correction and motion correction were also performed. The particles were imported into cryoSPARC (23) and were subjected to 2D classification, and *ab initio* reconstruction to generate an initial 3D model. Initial 3D models were subjected to multiple rounds of heterogeneous refinement to remove particles which did not represent Ku. The models’ resolution and density quality were further improved by performing homogeneous refinement and finally non-uniform refinement. The classification process is summarized schematically in Supplementary Figures 1–3. The reconstruction at 3.50 Å of Ku-IP6 contained 93 175 particles, and the 2.74 Å map for Ku-DNA-IP6 contained 276 013 particles. Final map resolutions were calculated in cryoSPARC (23) by Fourier shell correlation at 0.143 cut-off. For structure refinement, a previously published crystallographic model of Ku (PDB: 1JEY) was rigid body-fitted into both final cryo-EM maps using UCSF Chimera (24). Models were manually adjusted, refined and extra residues were built using Coot (25). The IP6 molecule (PDB: 7MOF) was also rigid-body fitted into the corresponding cryo-EM density using Coot (25). Several rounds of real-space refinement were then performed using PHENIX (26) until outliers were fixed. All final models were validated before being deposited into the PDB. The PDB codes for the cryo-EM structures of Ku 70-Ku80/IP6 and of Ku70–Ku80/DNA/IP6 are 7ZT6 and 7ZVT, respectively.

### Crystal structure

Crystallization screenings of the complex formed by Ku_ΔC_ heterodimer, a hairpin DNA (hDNA) used in (20,27) and IP6, were performed on the HTX platform (EMBL, Grenoble) by vapour diffusion with an automatic visualization at 4°C (28). Crystal hits were optimized manually in hanging drops at 20°C. hDNA was formed by hybridizing oligonucleotides 34mer-up and 21mer-down (see oligonucleotides in Supplemental Information). hDNA and IP6 were added at 1.2 molar excess to Ku_ΔC_ (after tag cleavage) dialyzed against 20 mM Tris pH 9, 500 mM NaCl, 5 mM TCEP. The optimized crystallization conditions are obtained by mixing 1.5 μL of protein complex at 12 mg/mL with 1.5 μL reservoir solution (24% PEG 3350, 200 mM Na_2_SO_4_, 100 mM BTP pH 8.5 and 5 mM β-mercaptoethanol). Crystals were cryo-protected in a mother solution supplemented with 20% v/v glycerol before freezing in liquid nitrogen. Diffraction data were collected at 100K on protein-crystallography beamline PROXIMA-2a (synchrotron SOLEIL, France) (Supplementary Table 2). Crystallographic data were processed with XDS (29) and scaled using Scala from the CCP4 suite (30). The crystals present a highly anisotropic diffraction (between 2.85 and 4.25 Å resolution according to the axes). The anisotropy of crystals was treated with the STARANISO program (http://staraniso.globalphasing.org/). Crystal structure was solved by Molecular Replacement with Molrep from the CCP4, by using the crystal structure of Ku (PDB: 1JEY). DNA and IP6 were observed in the electron density and positioned using Coot (25). The IP6 was positioned with the carbon in position 2 orientated towards solvent. It was indeed reported that an IP6 with a biotin attached in position 2 has still a stimulatory effect on c-NHEJ (31). Refinement was performed by BUSTER (32). In the final model, the following regions of Ku are not visible: Ku70 1–33 and 535–544 and Ku80 1–5, 176–180, 546–551. The quality of the model was assessed using PDB validation service One Deep. Data collection and refinement statistics for the crystal structure of Ku70–Ku80-DNA-IP6 are presented in Supplementary Table 2. The crystal structure of Ku70–Ku80/DNA/IP6 is deposited with the PDB code 7Z6O. Small-Angle X-ray scattering (SAXS) measurements were conducted at the SWING beamline (synchrotron SOLEIL, Saint Aubin). Data were recorded using an AVIEX170170 CCD detector with the same protocol used in (20).

### Total internal reflection fluorescence (TIRF) microscope setup

Single molecule labelling, and flow channel preparation for single-molecule colocalization experiment are described in Supplemental Information. A single-molecule experiment was performed on a Total Internal Reflection Fluorescence (TIRF) microscope equipped with an inverted oil immersion objective (×100, 1.49 NA lens) coupled to 532 nm (Ultra-Laser, MGL-FN-532) and 639 nm (Ultra-Laser, MGL-FN-639) excitation lasers to excite the sample at TIRF illumination. A dichroic beam splitter (Semrock, Di03-635-t1) was used to split green and red signals, which were passed through narrow bandpass filters (Semrock, FF01-582/64 and FF01-680/42). The signals were collected by an electron-multiplying charge-coupled device (EM-CCD) (Andor, iXon897). Fluorescence beads (Invitrogen, Life Technologies Corporation, Eugene, OR) were used to map corresponding signals in green and red channels.

### Single-molecule colocalization experiment

The BSA in the flow channel was washed with T50 buffer. 35 μL of 50 pM 18dsDNA construct was injected through the microfluidic channel, incubated for 3 min, and washed with T50 buffer. Imaging buffer (20 mM Tris, 100 mM KCl, 2.5 mM MgCl_2_ along with 1 mg/mL BSA, 0.8% glucose, 0.1% Trolox and 2 mM DTT adjusted to pH 7.5 added with 1% GLOX) containing 1 nM of AF647 labelled Ku protein was injected with and without 10 μM IP6 (Cat. P8810-10G, Sigma) through the channel and imaged in the TIRF microscope. Movies of 1 000 frames (33 Hz) were recorded with the red laser and both lasers turned on for the first 900 frames and last 100 frames, respectively. Four independent experiments were performed where at least 20 movies were recorded for each experimental condition. Signals from the red channel, which had corresponding signals in the green channel in the last 100 frames, were only selected based on the Matlab script mapping file. The time traces of the Ku-AF647 signals were fitted to a Hidden-Markov-Model (HMM) using the ebFRET package (33) to determine on and off dwell times. On and off states refer to the bound and unbound states of the Ku with 18dsDNA construct. The histograms of on and off dwell times were plotted. The plot was fitted with a single exponential decay function to calculate the *k*_ON_ and *k*_OFF_. The Student's*t*-test was performed.

### Cell lines and cell culture

U2OS human osteosarcoma cells (ECACC, Salisbury, UK) and HEK-293T human embryonic cells, were grown in DMEM (Eurobio, France) supplemented with 10% fetal calf serum (Eurobio, France), 125 U/mL penicillin, and 125 μg/mL streptomycin. Cells were maintained at 37°C in a 5% CO_2_ humidified incubator. Production of lentiviral particles in HEK-293T cells and transduction of U2OS cells were performed as previously described (34). The generation of U2OS-shKu80 cells expressing a doxycyclin-inducible shRNA against Ku80 has been previously reported (20).

### Laser micro-irradiation

Live cell microscopy and laser micro-irradiation were performed as previously described (34).

### Cell fractionation

Stock solution of Calicheamicin γ1 (Cali), gift from P.R. Hamann (Wyeth Research, Pearl River, NY, USA), was made at 40 μM in ethanol and stored at –20°C. For drug-exposure, exponentially growing cells in 60 cm diameter dishes were either mock-treated or treated with Cali in fresh medium for 1 h at 37°C. Then cells were washed with phosphate-buffered saline and trypsinized. Pellets were fractionated as follows. Cells were first resuspended for 7 min on ice in 120 μL of extraction buffer 1 (50 mM HEPES pH 7.5, 150 mM NaCl, 1 mM EDTA) containing 0.1% Triton X-100 and supplemented with the Halt protease and phosphatase inhibitor cocktail (Thermo Fisher Scientific) with intermittent gentle vortexing. Following centrifugation at 14 000 rpm for 3 min, the supernatant was removed and stored, then pellets were gently resuspended in 120 μL of extraction buffer 2 (50 mM HEPES pH 7.5, 75 mM NaCl, 1 mM EDTA) containing 0.025% Triton X-100 and 30 U RNAseA/T1 (Thermo Fisher Scientific), incubated for 15 min at 25°C under agitation and centrifuged as above. The supernatant was removed and mixed with the previous one, giving the soluble protein fraction. Pellets were resuspended in 120 μL lysis buffer (50 mM Tris pH 8.1, 10 mM EDTA) supplemented with 0.1% Triton X-100 and 0.3% SDS and sonicated (Vibracel, Bioblock Scientific) or passed 10 times through a 26G needle, giving the chromatin protein fraction. Protein content was measured with BCA reagent (Pierce) and equivalent protein amounts were denaturated in loading buffer at 1× final concentration (50 mM Tris–HCl pH 6.8, 10% glycerol, 1% SDS, 300 mM 2-mercaptoethanol, 0.01% bromophenol blue) at 95°C for 5 min, separated on SDS-PAGE gels (BioRad 4–15% TGX pre-cast gels) before overnight transfer onto Immobilon-P polyvinylidene difluoride (PVDF, Millipore) membranes. Staining with AdvanStain Iris (Advansta) controlled homogeneous loading and prestained protein ladder allowed cutting the membrane for separate and simultaneous blotting with selected antibodies. Membranes pieces were blocked for 60 min with 5% non-fat dry milk in PBS, 0.1% Tween-20 (Sigma-Aldrich) (PBS-T buffer), incubated as necessary with primary antibody diluted in PBS-T containing 1% bovine serum albumin (immunoglobulin- and lipid-free fraction V, Sigma-Aldrich) and washed 3 times with PBS-T; membranes were incubated for at most 1 hr with HRP-conjugated secondary antibodies in PBS-T and washed three times with PBS-T. Immuno-blots were visualized using autoradiography films together with enhanced chemiluminescence (WesternBright ECl, Advansta).

### Antibodies

For immunoblotting, were used: mouse monoclonal antibodies anti-beta-Actin (clone AC-15, Santa Cruz), anti-Ku70 (clone N3H10) and anti-DNA-PKcs (clone 18.2) (Thermo Fisher Scientific); rabbit polyclonal antibodies anti-XLF (A300-730A, Bethyl Laboratories or A199957, Abclonal), anti-LIG4 (A11432, Abclonal), anti-DNA-PKcs PhSer-2056 (ab18192), anti-PAXX (ab126353) (Abcam) and anti-γH2AX (Cell Signaling Technologies); Peroxidase-conjugated goat anti-mouse or anti-rabbit secondary antibodies were from Jackson Immunoresearch Laboratories.

### dEJ-HR reporter system

The dEJ-HR dual reporter substrate for both direct end-joining and homologous recombination was assembled into the pEGFP-N1 vector (Clontech) following sequential insertion of various PCR products, oligonucleotide linkers and synthetic DNA fragments (see Supplementary Information for a detailed description). The Cas9 and guide RNA co-expression plasmid used to cleave the reporter substrate was generated by inserting the pre-annealed gRNA-dEJ-HR-F and gRNA-dEJ-HR-R oligonucleotides into the BbsI restriction sites of pX330-U6-Chimeric_BB-CBh-hSpCas9 (a gift from Feng Zhang; Addgene plasmid # 42230; http://n2t.net/addgene:42230; RRID:Addgene_42230). The pmCherry-NLS plasmid (a gift from Martin Offterdinger; Addgene plasmid # 39319; http://n2t.net/addgene:39319; RRID:Addgene_39319) was used as an internal control of transfection efficiency in end-joining assays.

### Dual dEJ/HR assay

Cell lines and cultures are described in Figure 5 legend. HEK-293T cells were seeded in 6-well plates and transfected 24 h later with a mix of reporter substrate (500 ng), Cas9/gRNA expressing vector (300 ng) and pmCherry-NLS control plasmid (200 ng) using JetPEI (PolyPlus-Transfection, Illkirch-Graffenstaden, France). When indicated, cells were treated at the time of transfection with 3 μM DNA-PK inhibitor NU7441 (Tocris, Bristol, UK) or 10 μM Rad51 inhibitor B02 (Sigma-Aldrich St. Louis, MO). Cells were trypsinized 2 days post-transfection, washed with PBS and analysed by flow cytometry on a Fortessa X-20 cell analyzer (BD Biosciences). For each cell population, the integrated fluorescence signal (% positive cells × mean fluorescence) was calculated for each repair readout (i.e. GFP for direct end-joining and BFP for gene conversion) and normalized to that of transfection control (mCherry). Western blots, antibodies, oligonucleotides, plasmids and DNA manipulations, generation of U2OS shKu80 cells and generation of HEK-293T mAID-Ku70/mAID-Ku80 cells are described in Supplemental Information.

### Additional information

including reagents, oligonucleotides, mass spectrometry, biophysical approaches (calorimetry, switchSENSE and nanoDSF) and bionformatic analyses are described in Supplemental Information.

## Results

### Cryo-EM and X-ray crystallography structures of Ku reveal the binding site for IP6

To establish the structural basis of IP6 binding to the Ku heterodimer, we utilised both cryo-EM and X-ray crystallography in parallel. We first sought to optimise a method for the preparation of cryo-EM grids of both apo and DNA-bound full-length Ku (Ku_FL,_ MW 152 kDa). A Ku_FL_ expressed in Sf9 insect cells with a His-Tag at the N-terminus of Ku70 was used for these experiments_._ Data collected from these grids resulted in cryo-EM maps of apo- and DNA bound Ku_FL_ to 3.5 Å and 2.74 Å resolution respectively (Figure 1C, D, Supplementary Table 1, Supplementary Figures 1–4). Analysis of both maps revealed the presence of additional density at a site surrounded by residues previously reported to comprise the IP6 binding site (18) (Supplementary Figure 4f, g). As no exogenous IP6 was added to the protein sample, we hypothesised that IP6 bound Ku during expression of the complex in Sf9 insect cells and remained bound during the purification. To confirm whether the extra density observed corresponded to IP6, we analysed the purified Ku_FL_ sample by mass spectrometry. A peak at *m*/*z* 658.85Da was observed, consistent with the molecular weight of IP6 (Supplementary Figure 5).

**Figure 2.**
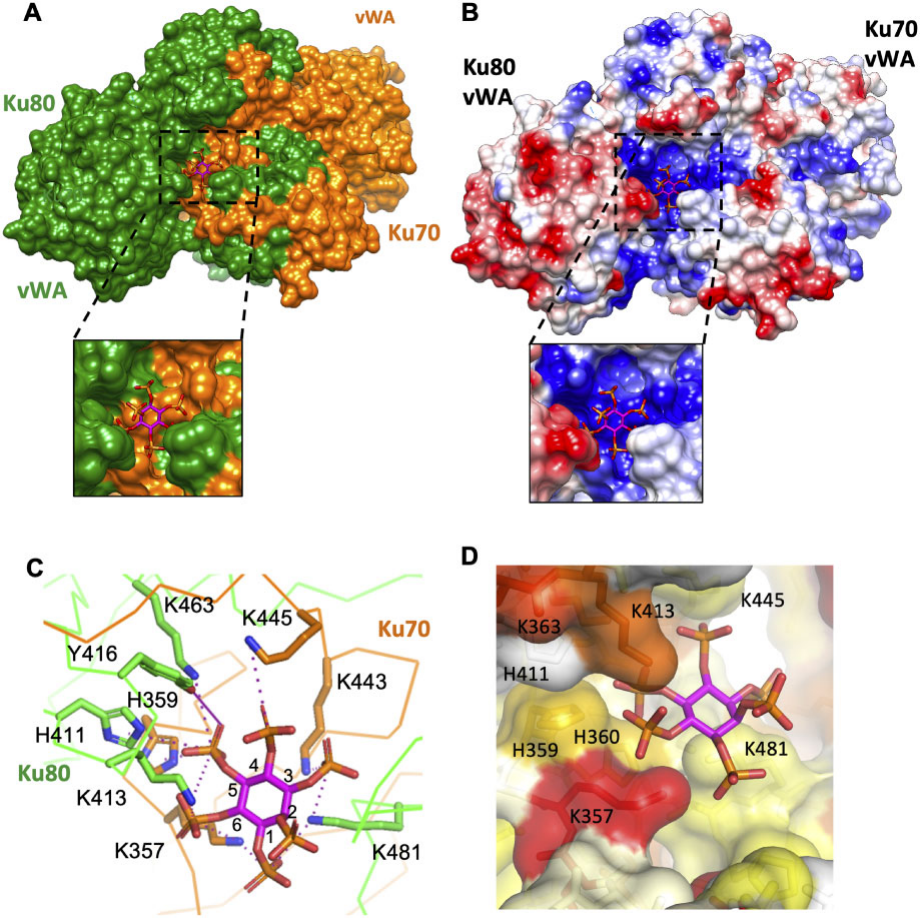
Structure of the IP6 binding pocket of Ku (**A**) IP6 (coloured by heteroatom, carbons: magenta, phosphorus: orange, oxygens: red) binds to a solvent exposed pocket at the interface between Ku70 (orange) and Ku80 (green). (**B**) Ku is shown as a surface and coloured according to its electrostatic potential. IP6 binds to a highly positively charged pocket at the surface of Ku. (**C**) Detailed visualisation of the IP6 binding pocket. The enumeration of the carbons is reported. Salt bridges and hydrogen bond are respectively in magenta dashed and plain lines (distances are presented in Supplementary Table 3). (**D**) Visualisation of the IP6 pocket coloured according to the conservation rate of the amino acids deduced from multiple sequence alignments of Ku70 or Ku80 eukaryotes sequences (Supplementary Figure 7). (Figure 2a,b and c, d are made with the cryoEM and X-ray structures of Ku-DNA-IP6, respectively).

**Figure 3.**
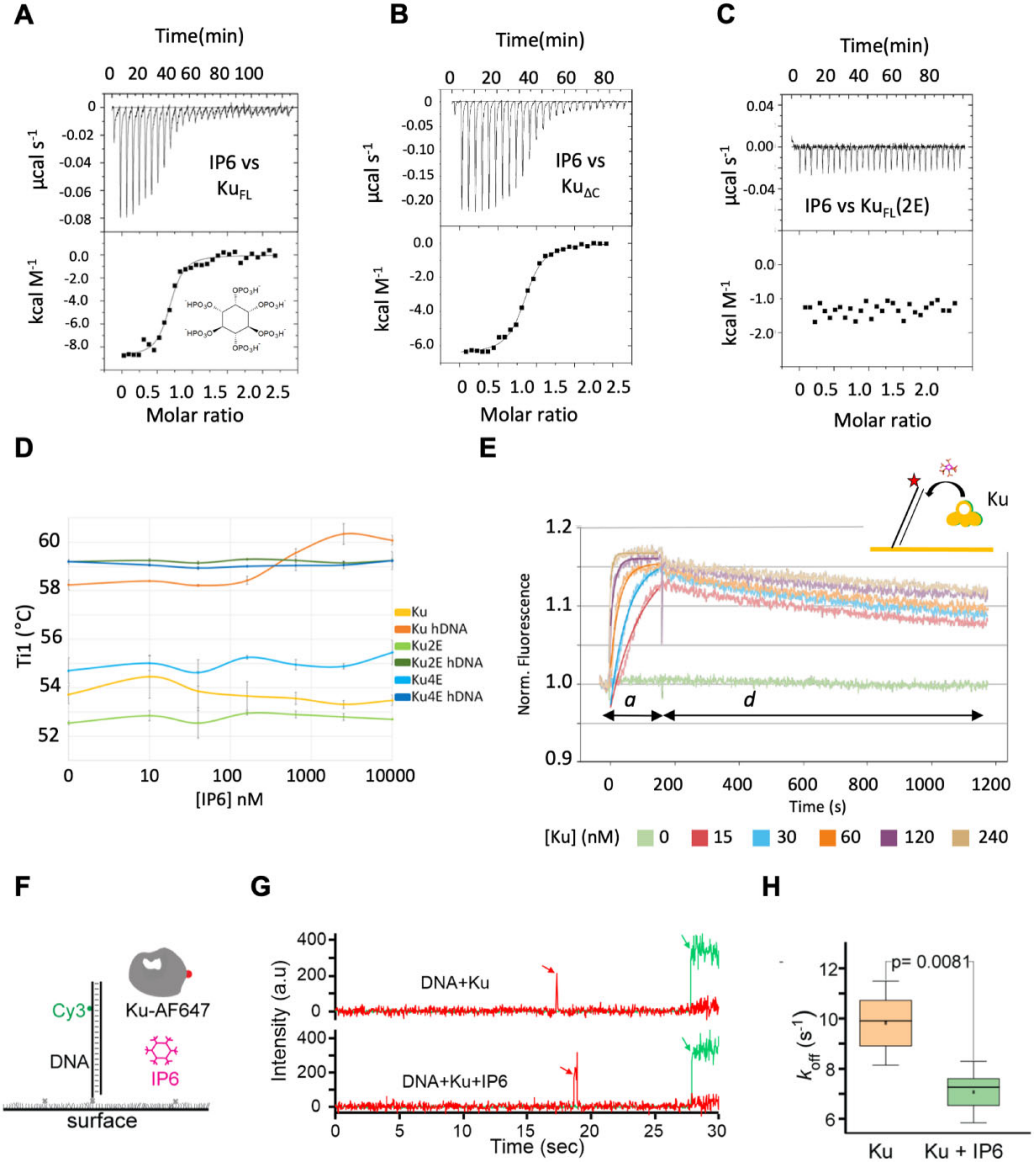
Impacts of IP6 on Ku stabilization and interaction with DNA. (**A–C**) Isothermal titration calorimetry (ITC) analyses of the interaction between (A) Ku_FL_ and IP6, (B) Ku_ΔC_ and IP6 and (C) the mutant Ku-2E (Ku70-K357E, Ku80-K481E) and IP6. All ITC experiments were done in duplicate. (**D**) Analysis of the thermal stability (Ti) by nano-differential scanning fluorimetry (nanoDSF) of Ku_FL_, Ku-2E and Ku-4E (Ku70-H359E and H360E)/Ku80-K413E and H414E), in presence or absence of a hairpin DNA as indicated and with IP6 concentration ranging from 0 to 10 μM. All nanoDSF experiments were done in triplicate. (**E**) switchSENSE kinetics analysis of the Ku interaction with a dsDNA (48 bp) bound to a chip. The experiments were performed in the presence of 3 μM IP6, with 3 min association time ‘a’ and 15 min dissociation time ‘d’, based on a duplicate experiment. Interactions were performed with Ku concentration ranging from 15 nM to 240 nM (see legend below x-axis). The same experiment was performed in parallel without IP6 (Supplementary Figure 10a). The fits of the association steps are superimposed on the raw data. (**F**) Principle of the single-molecule colocalization experiment. (**G**) Representative single-molecule time traces for Ku-AF647 binding on 18 bp DNA-Cy3 without (top) and with (bottom) 10 μM IP6. Red arrows indicate the binding of Ku-AF647 protein with DNA. The green laser is turned on at the end of the experiment, indicated by a green arrow, to confirm the presence of DNA. (**H**) Comparative *k*_OFF_ box plot of binding of Ku on DNA without (orange, *n* > 10 000) and with (green, *n* > 10 000) IP6, based on four independent experiments. *P* = 0.0081 indicates a significant difference between *k*_OFF_ without and with IP6 based on the Student's *t*-test. Error bars represent standard deviation.

**Figure 4.**
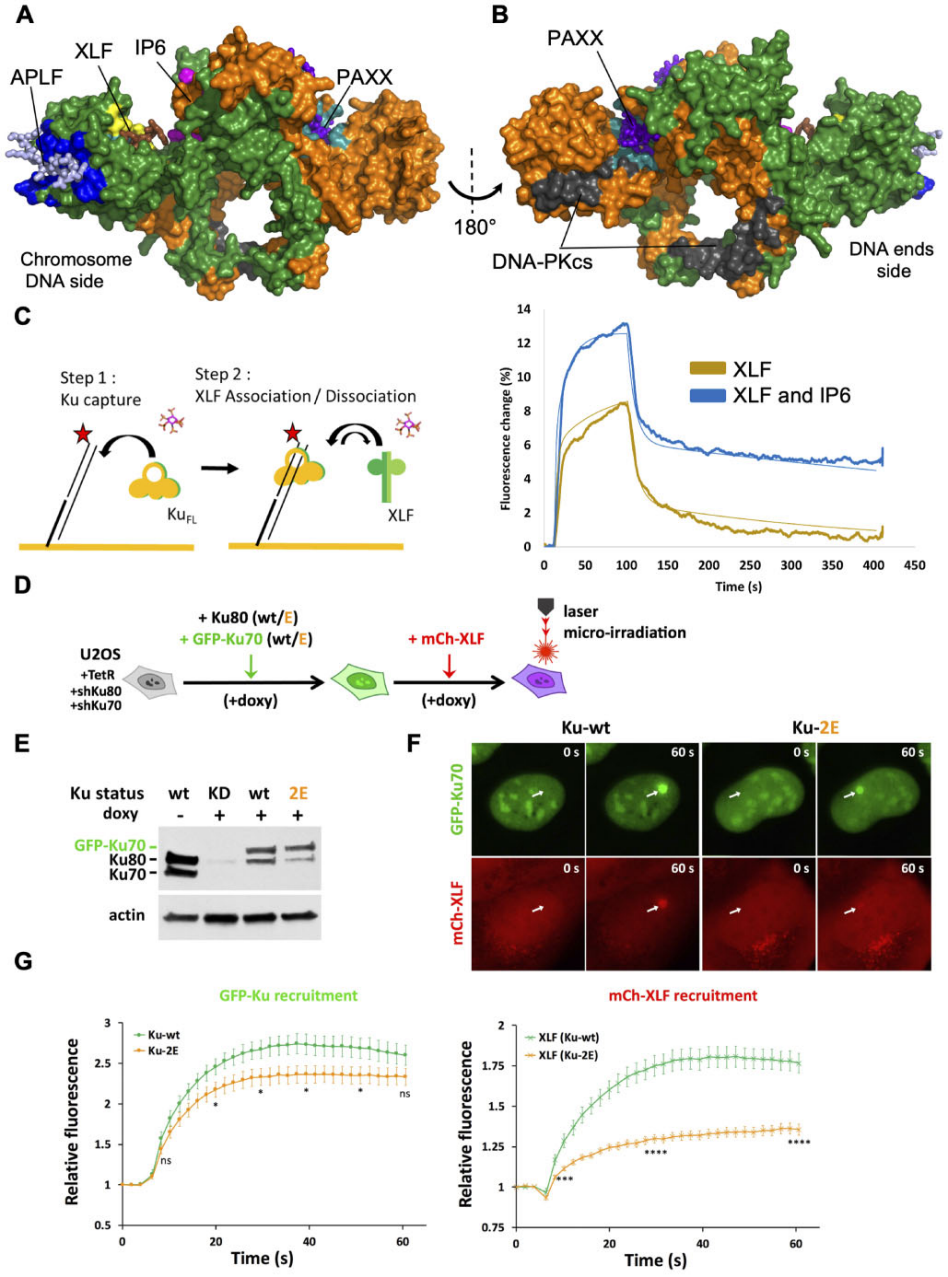
Effects of IP6 on Ku-XLF interaction and of combined Ku70 and Ku80 mutations on Ku and XLF recruitment to laser-induced DSBs in cells. (**A, B**) Position of IP6 (magenta) binding site relative to the binding sites on Ku of DNA-PKcs (grey). Interaction sites of Ku with XLF KBM (Ku site in yellow, XLF KBM in brown), APLF KBM (Ku site in dark blue, APLF KBM in light blue) and PAXX KBM (Ku site in cyan, PAXX KBM in violet) (KBM peptides are represented in stick). The XLF and PAXX KBMs bind respectively to Ku80 and Ku70 vWA internal position upon an opening of the vWA domains of each subunit. (The figure is made with the X-ray structure Ku-DNA-IP6 and the position of vWA domain of Ku70 observed in the X-ray structure with PAXX KBM (8BHY).) (**C**) switchSENSE analysis of Ku-DNA interaction with XLF (8 μM) in presence or not of 20 μM IP6 during both Ku association (step 1) and XLF titration (step 2). Results with additional concentrations of XLF are shown in Supplementary Figure 12. Binding curves in presence or absence of IP6 were fitted with a two steps binding mode (thin lines). Kinetics data are reported in a table in Supplementary Figure 12. (**D**) Schematic diagram illustrating the steps of cell lines construction. U2OS cells expressing Tet-On inducible shRNAs against Ku80 and Ku70 were complemented with lentiviral constructs allowing the expression of both Ku80-K481E mutant and GFP-tagged Ku70-K357E mutant (Ku-2E) or both wild-type Ku80 and wild-type GFP-tagged Ku70 (wt) as a control. Rescued cell populations were selected by doxycycline treatment (+doxy) and further transduced to express mCherry-tagged XLF (mCh-XLF). (**E**) Expression levels of Ku80 and Ku70 at each step of cell lines construction were assessed by western blot. (**F**) GFP and mCherry-fluorescent cells expressing either Ku-wt or Ku-2E (as in D) and mCh-XLF were then micro-irradiated. GFP-Ku and mCh-XLF recruitment at irradiated areas was measured. Typical images of fluorescent nuclei before and 60 s post-irradiation. The irradiated areas are indicated by arrows. (**G**) Quantification of fluorescence accumulation at laser-damaged sites over 1 min for U2OS cells expressing GFP-Ku-wt (green) or GFP-Ku-2E (orange) and mCh-XLF. Results were plotted as mean values ± standard error of the mean (SEM) from 19 (Ku-wt) and 20 (Ku-2E) individual nuclei. *P* values at various time points were calculated using unpaired two-tailed *t*-test. *P* values for GFP-Ku recruitment (Ku-wt vs Ku-2E) at 10, 20, 30, 40, 50 and 60 s were 0.1741 (ns), 0.0482 (*), 0.0319 (*), 0.0318 (*), 0.0396 (*) and 0.1073 (ns), respectively. *P* values for mCherry-XLF recruitment (Ku-wt versus Ku-2E) were < 0.001 (***) at 10 s and <0.0001 (****) at 30 and 60 s.

**Figure 5.**
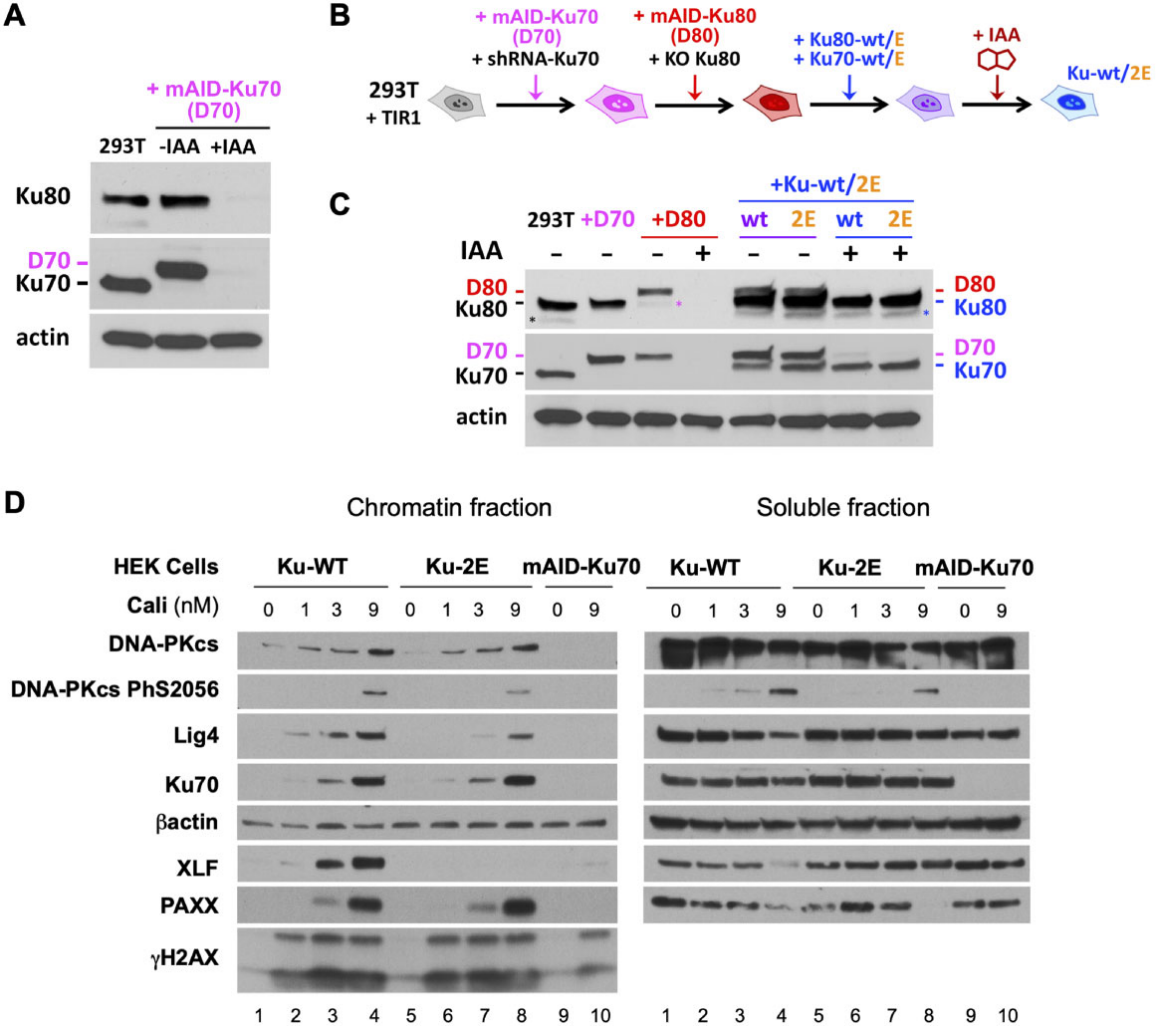
Impact of combined Ku70 and Ku80 mutations on c-NHEJ complex assembly in cells. (**A**) Western blot of cell extracts from HEK-293T cells showing mAID-Ku70/Ku80 expression levels before and after overnight treatment with auxin (IAA). (**B**) Schematic diagram illustrating the steps of cell lines construction and dual DSB repair (DSBR) reporter assay. In HEK-293T cells expressing OsTIR1, both Ku subunits were silenced (by shRNA expression for Ku70 and knock-out for Ku80, respectively) in order to replace endogenous Ku70 and Ku80 expression by degron-tagged constructs, mAID-Ku70 (D70) and mAID-Ku80 (D80), respectively. These cells are further transduced to express Ku-2E mutant (Ku70-K357E, Ku80-K481E) or wild-type forms as a control. Degron-tagged Ku subunits are then degraded upon addition of auxin (+IAA) leaving only Ku-2E or Ku-wt dimers. (**C**) Expression levels of Ku80 and Ku70 at the different steps of the cell lines construction. Asterisks in the Ku80 western blot indicate residual Ku70 signals from previous hybridization that resisted membrane stripping. (**D**) Western blot of chromatin and soluble fractions as described in Materials and Methods section, from HEK-293T cells expressing Ku WT or Ku-2E (as in B) or mAID-Ku70 (as in A), treated with IAA for 18 h and then treated or not with calicheamicin doses as stated for 1 h.

Models of apo and DNA-bound Ku were constructed using a previously determined X-ray crystallography structure of Ku as a starting model (PDB:1JEY) (27). All domains previously reported for Ku, including the vWA, β-barrel and ARM domains were similar in conformation and structure. We were able to build in a small loop region in Ku70 (residues 222–231) where density was previously missing. The similarity of these models with those previously published indicates that binding of IP6 does not induce any significant structural rearrangements in Ku. Due to their inherent flexibility, we did not observe additional density corresponding to the C-terminal region of Ku70 or Ku80. We modelled IP6 into the same area in both apo and DNA-bound Ku models (Figure 1C, D, Supplementary Figure 4f, g)).

In parallel to cryo-EM analysis, we performed X-ray crystallographic analysis of the Ku/DNA complex bound to IP6 (Figure 1E). For X-ray experiments, SAXS and biophysical measurements described in the rest of the article, we used Ku_FL_ expressed in Sf21 insect cells (and not in Sf9 as for cryo-EM). We also used a Ku construct (Ku70 1–544, Ku80 1–541) with both C-terminal regions deleted (Ku_ΔC_) also expressed in Sf21 insect cells (Figure 1A). These two last Ku heterodimers contain a His-tag at the N-terminus of Ku80 (and not at the N-terminus of Ku70 as for cryo-EM). We observed that in contrast to the Ku heterodimers prepared for cryo-EM, the Ku_FL_ and Ku_ΔC_ heterodimers thus produced did not contain IP6 after purification and thus allow to measure interactions with and without IP6 as well as the impact of IP6 on Ku heterodimer. This difference may rely on the position of the His-tag which is at the N-terminus of Ku70 (heterodimer used for cryo-EM and mass spectrometry) or at the N-terminus of Ku80 (heterodimer used for other analyses). We obtained crystals of Ku_ΔC_ in the presence of a hairpin DNA (as already used in (20)) and IP6 at a ratio of (1 Ku_ΔC_):(1.2 DNA):(1.2 IP6). Diffraction data were collected to 3.7 Å resolution at synchrotron SOLEIL on the PROXIMA-2a beamline (Supplementary Table 2). The structure was determined by molecular replacement using the same X-ray coordinates (PDB:1JEY) used for the cryo-EM models described above. The DNA was unambiguously positioned in the electron density and superimposes well with the DNA observed in the previous Ku-DNA structures (20,27). At this step, the highest electron density in the *mFo*-*DFc* map of the asymmetric unit was located in a basic pocket at the interface between Ku70 and Ku80 (Supplementary Figure 4h). We attributed this density to the IP6 molecule added during crystallization and performed an initial cycle of rigid body refinement followed by normal and TLS refinements. Superimposition of the two cryo-EM models on the crystallographic model showed a good alignment of the Ku structures (rmsd of 0.78 Å over 940 Cα) (Supplementary Figure 6a). The density of the IP6 molecule is located at the same location. Comparison of the electron density maps from the cryo-EM and crystal structures shows good complementarity of both methods. The electron densities of the six phosphates are slightly better defined in the X-ray maps while the electron density of the Ku side chains in contact with the IP6 are clearer in the cryo-EM maps. Combination of both maps allows to position the IP6 molecule and its six asymmetrical phosphates within the Ku site.

**Figure 6.**
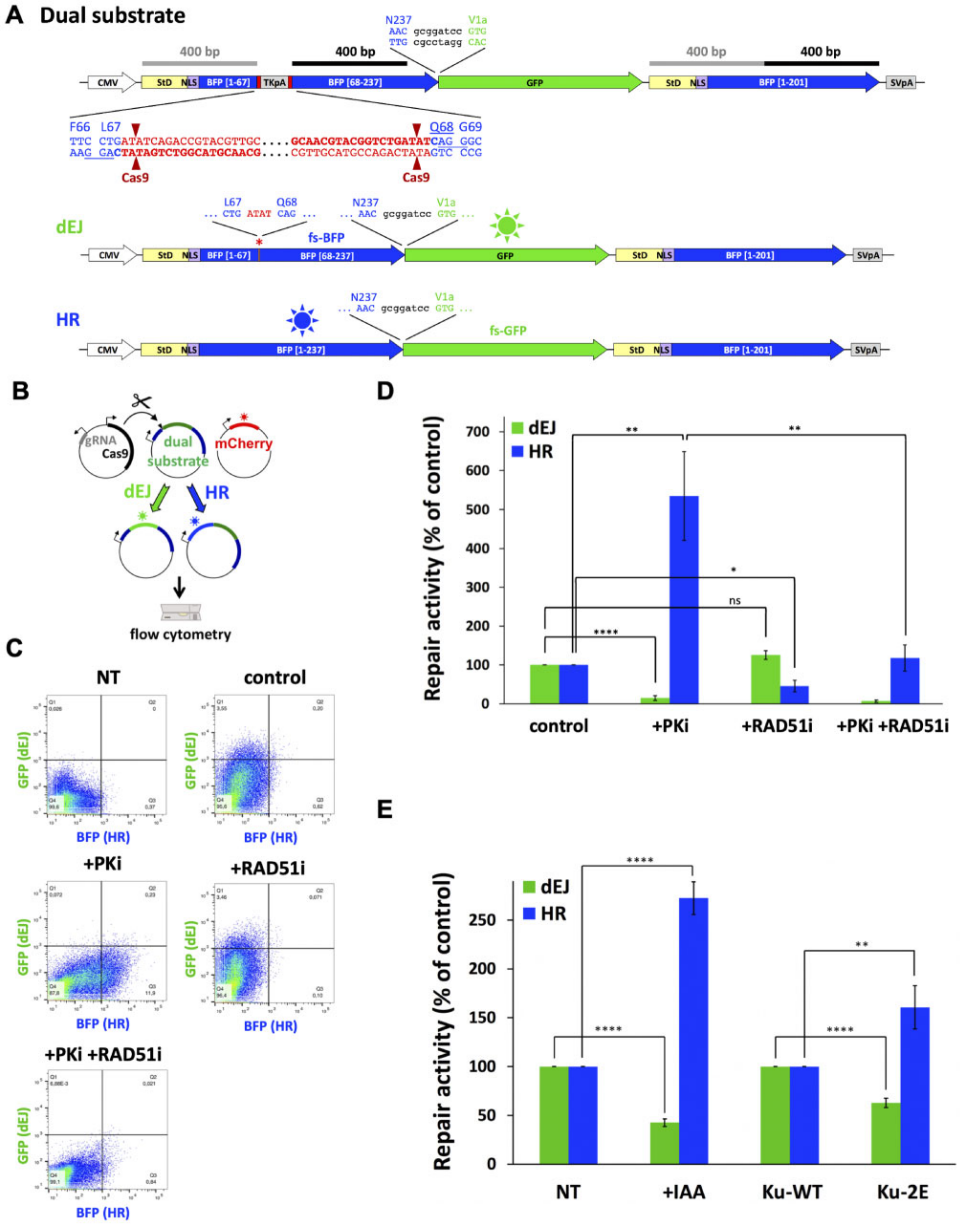
Impact of combined Ku70 and Ku80 mutations on End-Joining activity in cells. (**A**) The dual reporter substrate for both End-Joining and Homologous Recombination repair activities consists of two non-functional copies of mTagBFP2 cDNA (BFP) separated by an intervening EGFP cDNA (GFP). The first copy of BFP is interrupted near the chromophore coding sequence (between codons 67 and 68) by a cassette containing the HSV-TK polyadenylation sequence flanked by two inverted copies of a Cas9 target sequence (bold characters; the PAM AGG sequence is underlined). The downstream copy of BFP is truncated and stops at codon Y201. Following Cas9-mediated double cleavage, the HSV-TK polyadenylation sequence is deleted and the resulting DNA blunt-ends can be rejoined directly through NHEJ. This direct end-joining (dEJ) does not restore the BFP coding sequence because of a frameshift insertion (red asterisk and subsequent frameshifted BFP sequence (fs-BFP), but enables the expression of the downstream EGFP cDNA which lies in a different reading frame. In order to allow EGFP expression, all stop codons downstream the DNA cutting sites have been removed from BFP cDNA by silent mutations. Alternatively, the DSB can be repaired by gene conversion using the second BFP copy. A stuffer coding DNA fragment (StD) together with a nuclear localization sequence (NLS) have been fused upstream both BFP cDNA copies to provide 400 bp homology on both sides of the DSB, as indicated by grey and black lines, respectively. The homologous recombination event (HR) restores a full-length BFP cDNA whereas the EGFP cDNA lies in a different reading frame (fs-GFP) and is not expressed. (**B**) The dual DSBR reporter assay is performed by transfecting the dual reporter substrate together with a Cas9/gRNA-expressing vector to cleave the substrate and a mCherry-expressing vector to normalize for transfection efficiency. Fluorescence expression is analysed 48 h later by flow cytometry. Green (GFP) and blue (BFP) fluorescence represent direct end-joining (dEJ) and homologous recombination (HR), respectively. (**C** and **D**) Validation of the dual DSBR reporter assay in HEK-293T cells upon treatment with 3 μM DNA-PK inhibitor NU7441 (PKi) or 10 μM Rad51 inhibitor B02 (RAD51i). Results are presented as GFP versus BFP scatter plots of a representative experiment (C), or as a histogram with mean values ± SEM from 4–7 experiments (D). The control condition corresponds to a complete repair reaction without inhibitors. The P-values are as follows: < 0.0001 (****, dEJ, +PKi vs control), 0.1190 (ns, dEJ, + RAD51i versus control), 0.0089 (**, HR, + PKi versus control), 0.0332 (*, HR, + RAD51i versus control), 0.0076 (**, HR, + PKi/+ RAD51i versus + PKi). (**E**) Simultaneous measurement of direct end-joining (dEJ) and homologous recombination (HR) activities in HEK-293T cells depleted (+IAA) or not of Ku (Figure 5A) and in of HEK-293T cells expressing Ku-wt or Ku-2E (see Figure 5B) following two days post-transfection with the dual DSB repair reporter substrate. Results are normalized to 100% for the untreated respective control cells and presented as mean values ± SEM from X experiments. The *P*-values are <0.0001 (****, dEJ, +IAA versus –IAA, HR, +IAA versus –IAA and dEJ, 2E versus wt) and 0.0036 (**, HR, 2E versus wt).

Using limited proteolysis, Hanakahi *et al.* previously proposed that conformational changes may occur when IP6 binds to Ku in the absence of DNA, but that IP6 has no effect in the DNA bound form (16). The superimposition of the three Ku-IP6 complexes with or without DNA onto the Ku structures without IP6 (1JEY and 1JEQ) revealed no major conformational changes of the core regions. We could not exclude at this stage conformational changes of the C-terminal regions that are not visible in our cryo-EM and crystal structures. To further evaluate conformational changes including the C-terminal regions, we performed small angle X-ray scattering (SAXS) analyses of full-length Ku in complex with DNA in the presence or absence of IP6. The P(r) distributions indicate that the Ku_FL_-IP6 complex has a slightly larger Dmax value than the one measured with the Ku_FL_ heterodimer (Dmax measured are 206 and 201 Å respectively) (Figure 1F). The 5 Å variation may correspond to a small rearrangement in the presence of IP6 identified previously by limited proteolysis (16). Since this variation is far smaller than the 30 Å increase of Dmax reported for the Ku interaction with the XLF KBM and the corresponding large rearrangement of the vWA domain (20), we suggest that any conformational changes mediated by IP6 are modest.

### Characterisation of the IP6 binding pocket

The IP6 binding site is a shallow, solvent exposed pocket at the interface between the Ku70 and Ku80 subunits (Figure 2A). Surface of Ku coloured by electrostatic potential shows an extensive positive patch starting from the DNA binding site leading up to a highly positively charged pocket for IP6 binding (Figure 2B). Based on their calculated distances from the IP6 phosphate groups, we predict that residues K357, H359, K443 and K445 from Ku70 and K363, H411, K413, Y416 and K481 from Ku80 define the core of the binding pocket (Figure 2C). The IP6 binding site is located between the Ku70 and Ku80 β-barrel domains and the Ku80 N-terminal vWA domain. It is positioned on the face of Ku in contact with the chromosome DNA side rather than the face in contact with the DNA ends of the DSB. In a previous study, Cheung *et al.* (15) generated two Ku80 mutants (K238A/K239A and K233A/K238A/K239A) and one Ku70 mutant (K357A, K358A). All three mutants were affected for IP6 binding and impaired for *in vitro* NHEJ. K357 is the only residue in direct contact with IP6 (Supplementary Figure 6B). The four other positions are not in direct contact but in the vicinity, suggesting that these positions may contribute indirectly to IP6 binding.

We performed multiple sequence alignments to evaluate the conservation of the residues that delineate the IP6 pocket in eukaryotic Ku heterodimers and in bacterial Ku homodimers (Figure 2D, Supplementary Figure 7). Most of the residues of the IP6 binding pocket are well conserved in metazoan species, with the residues K357 of Ku70, K363 and K413 of Ku80 being the most highly conserved. Some of the residues within the binding pocket are conserved mainly in vertebrates (H359 of Ku70, Y416 and K481 of Ku80). We observed that the IP6 binding pocket is less conserved outside metazoan organisms (Supplementary Figure 7). The IP6 pocket is not conserved in fungal Ku or in bacterial Ku despite IP6 being present in these organisms. This observation is in good agreement with the absence of interactions reported between *S. cerevisae* Ku and IP6 (16).

### Specificity of the Ku binding site for IP6 and IP6 analogs

We then measured by isothermal titration calorimetry (ITC), the affinity of Ku_FL_ for IP6 and observed an interaction with a *K*_D_ of 3.5 ± 2.9 nM (Figure 3A). The interaction is enthalpy driven in good agreement with the electrostatic contacts observed in the cryo-EM and crystal structures. Previous competition assays performed with ^3^H-IP6 showed a *K*_D_ of 46 nM (18). We recently showed that the C-terminal region of Ku70 can be positioned in the region of IP6 pocket (35). We then analysed the affinity between the Ku_ΔC_ construct and IP6 and measured a ∼50-fold weaker affinity with a *K*_D_ of 178 ± 12 nM (Figure 3B). This suggests that the C-terminal regions of Ku though not visible in both cryoEM and crystal structure contribute to Ku interaction with IP6.

The IP6 molecule has six phosphates positioned in a precise orientation relatively to the planar carbon ring. We analysed the affinity of different stereoisomers of the IP6 to evaluate the importance of the relative position of the phosphate atoms. We measured the affinity of two synthetic analogues of the IP6, the scyllo-IP6 and the D-chiro-IP6 that differ from the natural IP6 by the spatial positions of the phosphate groups (Supplementary Figure 8a, b). We measured an affinity slightly weaker for the scyllo-IP6 and D-chiro-IP6 with *K*_D_ values respectively of 18 ± 2.5 and 22 ± 6.8 nM suggesting that the Ku binding pocket has a higher complementarity for the natural IP6 stereoisomer compare to two other IP6 analogs.

### IP6 stabilizes the Ku heterodimer in a DNA dependent manner

We then evaluated the impact of IP6 binding on the thermodynamic stability of Ku or Ku mutants using nanoDifferential Scanning Fluorimetry (nanoDSF). To impair IP6 binding, we chose to mutate two positions on the same side of the IP6 binding site (K357 on Ku70 and K481 on Ku80) (Figure 2C), that were both changed to glutamic acid (Ku-2E mutant) (Supplementary Figure 8c) and we purified the corresponding Ku-2E protein (Supplementary Figure 8d). As expected, this mutant showed no interaction with IP6 as measured by ITC, in comparison to Ku wild type (Figure 3C). Firstly, we measured the thermal stability of Ku_FL_ and Ku_ΔC_ and observed that the C-terminal regions contribute to a stabilization of Ku heterodimer by ∼3°C (Supplementary Figure 8e). We then analysed the stabilization effect of DNA on Ku using a hairpin DNA and measured a stabilization by 4–5°C (Figure 3D, compare values of Ti at 0 nM IP6 of Ku (light orange) and Ku-DNA (dark orange). We then analysed the effect of IP6 by measuring the thermal transition with increasing concentrations of IP6 from 10 to 10 μM. We observed no significant effect on Ku in the absence of DNA. In presence of DNA, Ku stability increased up to 2°C at the highest IP6 concentrations (Figure 3D). The Ku-2E mutant presents a thermal stability similar to that of Ku wild type as evaluated by nanoDSF and is stabilized by DNA in a similar manner, but no extra stabilization by IP6 was observed (Figure 3D, Supplementary Figure 9a, b). A Ku-4E quadruple mutant was also constructed and purified, bearing mutated positions different from that in Ku-2E (Ku70 H359E-H360E/Ku80 K413E H414E) (Supplementary Figure 8c, d). This 4E mutant was also stabilized by DNA in a similar manner as 2E mutant and wild type control but again was not further stabilized by IP6 (Supplementary Figure 9b). And as expected, this new mutant showed no interaction with IP6 as measured by ITC (Supplementary Figure 9c). Finally, we observed the same effect with the IP6 analogues albeit at higher concentrations due to their weaker affinity (Supplementary Figure 9d).

### IP6 increases Ku residence on DNA

We then investigated the impact of IP6 on the Ku-DNA interaction. We first used switchSENSE, a recent surface-based method which uses nucleotide nanolevers labelled with a fluorescent probe (36). The interaction between Ku_FL_ and a 48bp DNA bound on the chip, gave a *k*_ON_ of 6.94 ± 0.16 10^5^ M^−1^ s^−1^, a *k*_OFF_ of 2.26 ± 0.03 10^−3^ s^−1^ and a *K*_D_ of 3.25 ± 0.09 nM in agreement with the values measured by other biophysical approaches (37,38) (Supplementary Figure 10a). We then repeated the experiment with Ku_FL_ pre-incubated with an excess of IP6 (3 μM) and observed a slight stimulation of the interaction (*k*_ON_ = 6.35 ± 0.13 10^5^ M^−1^ s^−1^, *k*_OFF_ = 1.27 ± 0.03 10^−3^ s^−1^ and a *K*_D_ = 1.99 ± 0.06 nM) (Figure 3E).

Then we used a single-molecule FRET method with a Ku_FL_ construct fused with a SNAP tag to label Ku with a Cy5 fluorescent probe and immobilized DNA on a PEG surface (Figure 3F). Single molecules traces of the interaction were measured with or without IP6 at a concentration of 10 μM (see Figure 3G for a representative single-molecule time traces for Ku binding on 18bp DNA without and with IP6 and Supplementary Figure 10b for additional examples of traces). Ku has longer residence (red trace) on DNA immobilized in presence of IP6. Traces were fitted with the HMM model to get on and off dwell times. The single molecule FRET analysis shows that the presence of IP6 slightly increases the residence times of Ku on DNA and thus decreases the kinetic dissociation constant (k_OFF_) of Ku from dsDNA in agreement with switchSENSE measurements (Figure 3H and Supplementary Figure 10c).

### IP6 stabilizes XLF-Ku interaction *in vitro*

Even though the proposed IP6 binding site is in close proximity to the DNA binding site, none of the IP6 binding residues are directly involved in DNA binding (Figure 1D, E). In addition, when comparing the position of the IP6 binding pocket with respect to the structurally defined interaction sites of Ku partners it appears that IP6 is not located in close proximity to most of the known protein interaction sites on Ku. Indeed, the DNA-PKcs binding site is located towards the DNA ends on the opposite face of Ku (5,39) (Figure 4B, Supplementary Figure 11a, b) and the binding site of the APLF-like KBM (present in APLF, WRN and Cyren) (4,40), and of that of PAXX that we recently characterized (41) are located at distance in the Ku80 and Ku70 vWA domains, respectively (Figure 4A). In contrast, the IP6 binding pocket is in close proximity to the XLF KBM binding site (Figure 4A, Supplementary Figure 6c) (20). A residue from Ku70, K445 that interacts with the XLF KBM (PDB 6ERG) makes contacts in the structures reported in this study with IP6 (Supplementary Figure 6c). When the Ku crystal structure bound to the KBM peptide of XLF (PDB: 6ERG) was docked onto our cryo-EM models of NHEJ supercomplexes containing XLF and IP6, the density corresponding to the XLF N-terminus appeared close to the IP6 binding site (Supplementary Figure 11c). To evaluate the impact of IP6 binding on Ku-XLF interaction, we hybridized fluorescent DNA on the gold surface of a switchSENSE chip, bound Ku to this DNA in saturating conditions and then injected XLF as previously described (20) (Figure 4C, Supplementary Figure 13). The experiment was performed using three concentrations of XLF (1, 2 and 8 μM) in absence or presence of IP6 at 20 μM (Figure 4C and Supplementary Figure 12). We observed a biphasic interaction in both cases. The two apparent affinities between Ku and XLF when IP6 was present (*K*_D1_ = 1.2 ± 0.15 μM, *K*_D2_ = 0.13 ± 0.009 μM) were 3- and 14-fold stronger than the one observed in absence of IP6 ((*K*_D1_ = 3.6 ± 0.6 μM, *K*_D2_ = 1.9 ± 0.8 μM). These data indicate a stabilization of the Ku-XLF interaction by IP6.

### Ku and XLF recruitment at DSB sites are reduced by mutations in the IP6 binding site

We took advantage of the Ku-2E and Ku-4E mutations on different sites that similarly impair IP6 Ku binding (Figure 3C and Supplementary Figure 9c) to assess the impact of Ku lacking IP6 on various readouts for c-NHEJ complex assembly and activity in cells. Notably, Ku70–Ku80 mutated positions in the IP6 binding site are remote from the XLF binding site on Ku80 (Supplementary Figures 6c and 8c). Firstly, we analysed by live cell imaging the recruitment of Ku at DSB sites induced in nuclei by laser micro-irradiation, as previously described (20). We used U2OS cells expressing inducible shRNA against Ku80, allowing Ku80 replacement by wild type (wt) or mutated forms (20), followed by transduction with wild-type (wt) or mutated GFP-Ku70 constructs (Supplementary Figure 13a). In cells, Ku-2E exhibits similar expression level and nuclear pattern as Ku-wt (Supplementary Figure 13b, c). When we monitored GFP-Ku70 accrual at irradiated sites, we observed a decreased recruitment of Ku-2E to damaged sites compared with Ku-wt (Supplementary Figure 13c, d). The Ku-4E mutant also displayed a decreased recruitment to damaged sites (Supplementary Figure 13e). However, these cells express endogenous Ku70 which may compete with the GFP construct. Therefore, we repeated the experiment in U2OS cells in which both Ku subunits were silenced by doxycyclin-inducible shRNA expression against Ku70 and Ku80, and replaced by shRNA-resistant wt or mutant Ku subunits, Ku70 being fused to GFP. Due to the cell lethality resulting from Ku depletion upon doxycyclin addition, the survival of these cells relies on the expression of both Ku70 and Ku80 transduced constructs, either wt or mutated (Figure 4D, E). In this improved cell model, the recruitment of the GFP-Ku70 Ku-2E mutant was still decreased as compared to cells expressing wild-type Ku, especially at early time points (Figure 4G).

Then, we challenged in cells whether the binding of IP6 to Ku impacts on XLF enrolment in NHEJ as observed with purified proteins (Figure 4C). We took advantage of the auxin-mediated degradation of Ku to validate that XLF recruitment was fully Ku-dependent (Supplementary Figure 13f). Under these conditions, we measured the simultaneous recruitment of mCherry-XLF and GFP-Ku70 at laser sites in cells expressing Ku-wt or Ku-2E (Figure 4F, G). XLF recruitment was strongly impaired in cells expressing Ku-2E, to an extent much greater than that of the Ku mutant itself. This defect in XLF accrual at damaged site cannot be explained by the slight defect in Ku recruitment, since PAXX recruitment, while being strictly Ku-dependent (41), was even slightly enhanced in cells expressing Ku-2E (Supplementary Figure 13g), in sharp contrast with XLF (Figure 4G).

We challenged these results with an orthogonal method and in a different cell model. Cell fractionation in HEK-293T was performed following treatment with the strong clastogenic molecule calicheamicin, allowing detection of stable recruitment of c-NHEJ proteins at broken chromatin, as previously reported (42). HEK-293T cells were engineered to express mAID-Ku70 (Figure 5A, B) or both mAID-Ku70/80 (Figure 5A–C) and were either not complemented or complemented with wt or mutated Ku forms. Thus, auxin treatment established conditions for either no Ku expression (Figure 5A) or expression of Ku-wt or Ku-2E at similar levels (Figure 5C). Upon damage infliction to chromatin with increasing concentrations of calicheamicin in HEK-293T cells expressing Ku-wt, all the NHEJ proteins tested including XLF and PAXX were increasingly detected in the chromatin fraction containing the DSB marker γH2AX (Figure 5D, chromatin fraction, lanes 2–4). Moreover, XLF, Lig4 and PAXX were depleted from the soluble proteins fraction at the highest dose (Figure 5D, soluble fraction, lane 4). As expected from the hub function of Ku in c-NHEJ (4), none of these proteins was mobilized to the chromatin in the absence of Ku (Figure 5D, chromatin fraction, lane 10). Strikingly, with the Ku-2E mutant, XLF was not detected in damaged chromatin while Ku, DNA-PKcs and PAXX were recruited (Figure 5D, chromatin fraction, lanes 6–8), the latter protein being still depleted from the soluble fraction at the highest dose, in contrast to XLF (Figure 5D, soluble fraction, lane 8). As previously reported (43), the defect in XLF recruitment destabilized in turn the recruitment of Lig4 and interfered with end-synapsis accounted by the auto-phosphorylation of DNA-PKcs on S2056. An identical selective defect in XLF accrual at damaged chromatin was observed in cells expressing the Ku-4E mutant (Supplementary Figure 13h). Collectively, these data support that in cells, IP6 binding to Ku modulates Ku retention at DNA damage sites and XLF enrolment in the c-NHEJ complex.

### End-joining activity is compromised by mutations in the IP6 binding site

We then assessed the impact of the Ku-2E mutation on end-joining (EJ) activity in HEK-293T cells (more suitable for transfection experiments than U2OS cells). We designed a Cas9-targeted two-pathway reporter plasmid transfected in cells to quantify simultaneously the contribution of EJ and homologous recombination (HR) to the repair of a double DSB (Figure 6A). The reporter is designed such that following Cas-9-induced DSBs, repair events cause restoration of BFP or GFP sequences, and expression of GFP and BFP fluorescent proteins is measured by flow cytometry. Expression of m-Cherry from a co-transfected plasmid accounts for transfection efficiency (Figure 6B). Using classical inhibitors of either DNA-PK catalytic activity (NU7441, PKi) (44)) critical for EJ, or of the RAD51 HR protein (B02, RAD51i) (45) we observed that PKi decreased the expression of the direct EJ (dEJ) marker GFP while RAD51i decreased the expression of the HR marker BFP (Figure 6C, D). In addition, we measured a compensatory increase in HR upon dEJ inhibition through the use of PKi, as reported in Cas-9-mediated genome editing (Figure 6C, D, (46,47)). Moreover, we showed previously that deletion of c-NHEJ genes compromised GFP repair events (41). Despite the product plasmids were not sequenced, these data collectively support that expression of GFP and BFP accounts for dEJ and HR activities respectively. Then, we used HEK-293T cells under conditions for either no Ku expression or expression of Ku-wt or Ku-2E at similar levels (see Figure 5A–C). Interestingly, Ku depletion upon auxin addition impaired dEJ and increased HR on the Cas9-targeted plasmid as did PKi addition, further validating this system as a suitable dual reporter for monitoring both dEJ and HR (Figure 6E). When compared to cells expressing Ku-wt, Ku-2E mutant cells exhibited a 40% dEJ deficiency, mirrored by a significantly increased HR activity (Figure 6E).

Altogether, these data support that modulation of Ku retention at DNA damage sites and XLF enrolment in the c-NHEJ complex through IP6 binding to Ku have an impact on EJ efficiency in cells.

## Discussion

Ku has a central role in the c-NHEJ pathway through its ability to recognise DNA ends and to act as a hub for the recruitment of several key repair factors (4,48,49). The IP6 molecule was shown two decades ago to stimulate the c-NHEJ pathway (15) and molecular modelling and site directed mutagenesis proposed a first broad delineation of an IP6 binding pocket on Ku (18). In the present study, we combined cryo-EM and X-ray crystallography to characterize the IP6 binding site, which we located at the interface between Ku70 and Ku80. Then, we characterized the multiple impacts of IP6 on Ku function: neutralization of a highly positively charge pocket, increase in the DNA-dependent thermal stabilization of the heterodimer Ku, increase of the interaction between Ku-DNA and XLF *in vitro*, modulation of the Ku association to DSB sites and of Ku-dependent XLF engagement in the c-NHEJ complex, and subsequently control of repair activity in a cellular context.

This study is an example of the strength of adopting a multidisciplinary approach, since the two structural techniques we used complement each other. Our cryo-EM models provided an overall better quality of the density of the Ku residues of the IP6 binding site whereas the density for the IP6 molecule was better defined in the crystal structure. The negatively charged phosphates of IP6 have a negative contribution to the coulomb potential map generated by the cryo-EM experiment. In addition, the map can also be affected by enhanced radiation damage to these groups (50). The significant improvement in resolution observed for the Ku-DNA model, which refined the local resolution of the lysine side chains and coulomb potential for IP6, could be partly attributed to PEGylation of the sample prior to vitrification, following recent work by Zhang *et al.* (51). PEGylation of protein samples for cryo-EM analysis can improve their stability and protect them from denaturation or aggregation effects, therefore resulting in improvement in particle quality (51).

Noteworthy, IP6 was naturally bound to Ku in both our cryo-EM models, in contrast to our crystallographic structure in which IP6 was co-crystallized. We believe that such difference is due to location of the 10x His-tag on the Ku80 N-terminus, close to the IP6 binding site, in the Ku construct used for the crystal structure. Therefore, during the Nickel purification step, where the 10x His-tag interacts with the column, the Ku-IP6 interaction could be disrupted.

We showed that IP6 binds to a highly positively charged pocket at the Ku heterodimer interface, consisting mainly of lysine and histidine residues. The majority of the interactions are electrostatic, with the lysine side chains forming salt bridges with the phosphate groups, while histidine residues could potentially stabilise IP6 binding through planar interactions with its central carbon ring. Notably, we also observed density for IP6 in the same location in all our recently obtained high-order NHEJ supercomplexes (Supplementary Figure 11) (5,48). Recently, Liu *et al* reported a cryo-EM structure of Ku in complex with DNA-PKcs, Artemis and DNA (19) and observed an IP6 molecule in the same binding pocket (Supplementary Figure 6d–f). Comparison of the phosphate positions was challenging due to an atypical stereochemistry of the phosphate in the IP6 in Liu's study. In all the cryoEM studies, the IP6 molecule was not added to the cryo-EM samples but co-purified with Ku. This indicates a high stability of the complex supported by the affinity in the nanomolar range of IP6 for Ku that we measured by ITC. Since the intracellular concentration of IP6 is in the micromolar range (52), this also supports a physiological relevance of a Ku-IP6 complex which is most likely the form of Ku that initially binds to DSB ends in cells and that persists along the NHEJ supercomplexes assembly.

Several studies reported roles of IP6 as a co-factor for proteins or complexes involved in different cellular processes, from yeast to human. For example, in modulation of cell-growth and UV-induced apoptosis, IP6 has been shown with nanomalor potency to regulate ubiquitylation activity of cullin-RING E3 Ligases by stimulating physical interaction with the COP9 signalosome and thereby the assembly of an inactive complex (53). Most often the metabolite unexpectedly co-purified with the proteins expressed in eukaryotic cells (13,14). All IP6 binding pockets including Ku have high similarities with a large number of basic residues involved in binding of the metabolite, as expected. With the exception of ADAR2 (13), the binding pocket is solvent exposed, which could be necessary for the fast association and dissociation of IP6 in a cellular context.

The IP6 pocket in Ku is ∼20 Å remote from the entrance of the DNA binding site. Stability measurements by nanoDSF show an increased stability of Ku upon DNA binding and a further increase upon IP6 binding. Our recent cryo-EM models of NHEJ supercomplexes (48) illustrate that IP6 is present on Ku together with XLF, indicating that although their binding sites are close, their interactions with Ku are not exclusive. Finally, we showed by ITC that Ku interaction with IP6 is stabilized by the C-terminal regions of Ku suggesting that conversely the presence of IP6 may influence the positioning of Ku C-terminal regions.

We showed that abolishing IP6 interaction with Ku through mutagenesis of the binding pocket impacted on Ku accrual at damaged sites, XLF enrolment in the c-NHEJ complex and subsequent end-joining activity in cells. These data on cellular end-joining nicely corroborate previous observations with biochemical end-joining assays (15,16,18). Mutated Ku positions in the IP6 binding site used here were remote from the XLF binding site and similar results were obtained with two batches of different mutations (Ku-2E and Ku-4E). Nevertheless, we cannot exclude that the effects we measured on XLF were due to an impact of the mutations *per se* rather than to the lack of IP6 binding. However, we believe this is highly unlikely for several reasons. First, we demonstrated with purified proteins that IP6 stabilizes XLF interaction with Ku. Second, experiments *in vitro* with human cell extracts showed that IP6 stimulated concomitantly end-joining of a linear plasmid substrate and DNA-PK dependent phosphorylation of XLF (54). Since both DNA-PK auto-phosphorylation during the end-joining reaction and DNA-PK activity on a peptide substrate were insensitive to IP6, it was concluded that the effect of IP6 was specific for XLF. Moreover, stimulation of DNA-PK dependent phosphorylation of XLF required IP6 association to Ku (54). As suggested by the authors, the most likely interpretation of these data is that IP6 influences XLF interaction with Ku, and thereby the accessibility of XLF to phosphorylation by DNA-PK, in good agreement with our *in vitro* and cellular data.

The function of Ku in c-NHEJ is regulated by several post-translation modifications (3). Phosphorylation by DNA-PKcs is reported to increase Ku dissociation from DNA ends *in vitro* and prevention of these phosphorylation events in cells are not required for c-NHEJ (55) but rather impairs HR (56). Poly-(ADP-ribosylation) by PARP-1 was shown to lead to a decreased affinity of Ku for DNA (57). Ubiquitination by E3 ligases initiates Ku extraction by an AAA + ATPase after Ku is trapped on DNA following ends ligation (58). IP6 is to our knowledge the only Ku cofactor with a stimulatory role on c-NHEJ. Additional studies should help to understand in which context stimulation by IP6 may have a physiological role, in particular on DNA repair and whether such effect is associated with variations on the level of IP6 in cells as proposed for other proteins that use IP6 as cofactor (13).

## Supplementary Material

gkad863_Supplemental_Files

## Data Availability

The data underlying this article are available in the Protein Data Bank at https://www.rcsb.org/, and can be accessed with IDs 7ZT6 (cryoEM Ku-IP7), 7ZVT (cryoEM Ku-DNA-IP6), and 7Z6O (Xray structure Ku-DNA-IP6).
