# Supplementary material for: Structural and functional basis of inositol hexaphosphate stimulation of NHEJ through stabilization of Ku-XLF interaction
Source: Nucleic Acids Res. 2023 Oct 23;51(21):11732–47. doi: 10.1093/nar/gkad863 (PMC10682503; doi:10.1093/nar/gkad863)
Supplement: gkad863_Supplemental_Files [file gkad863_supplemental_files.zip › Ku-IP6 SI Fig1-13 v17_PC without red.pptx]

## Slide 1
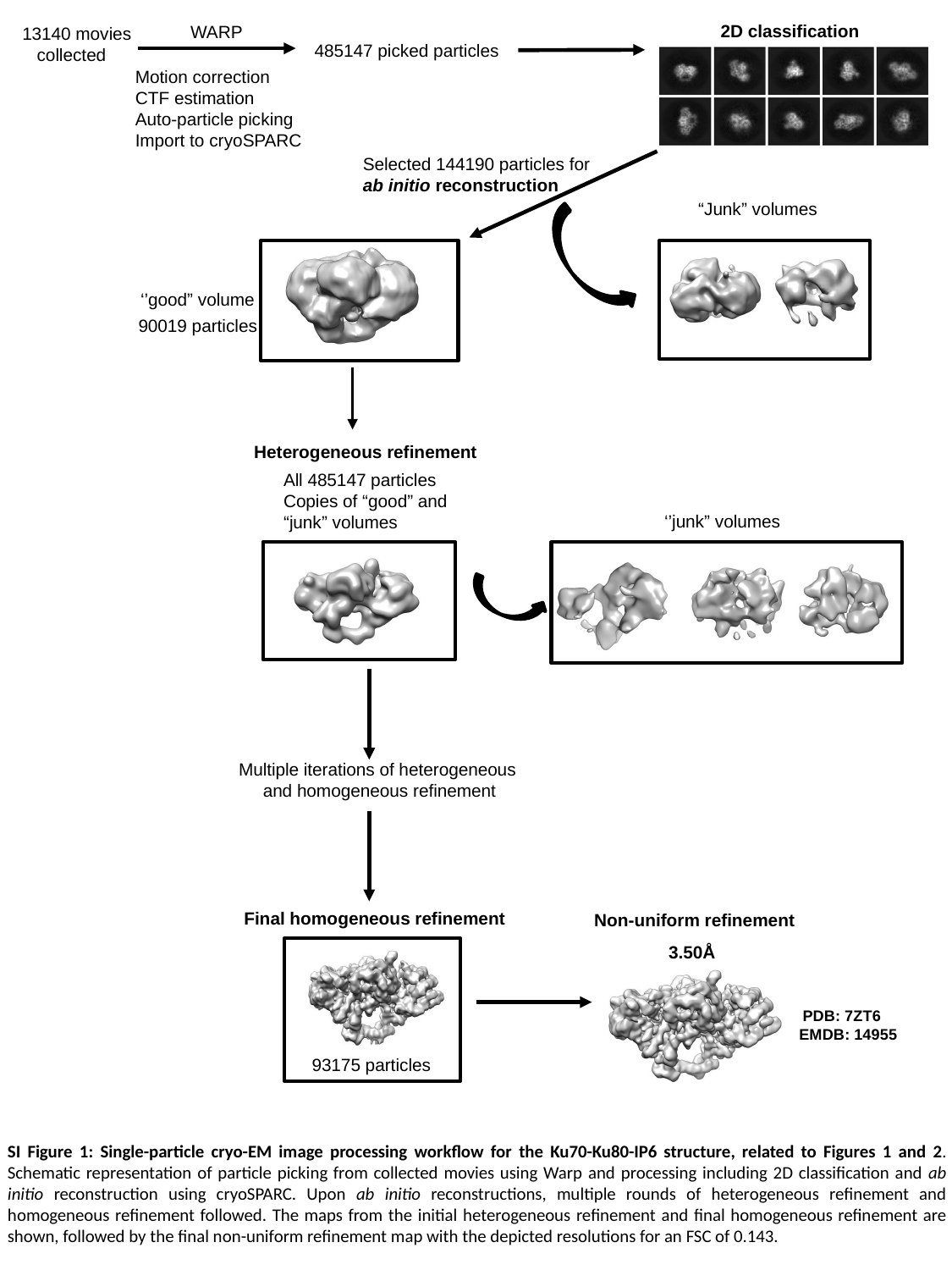

2D classification
WARP
13140 movies
 collected
485147 picked particles
Motion correction
CTF estimation
Auto-particle picking
Import to cryoSPARC
Selected 144190 particles for ab initio reconstruction
“Junk” volumes
‘’good” volume
90019 particles
Heterogeneous refinement
All 485147 particles
Copies of “good” and “junk” volumes
‘’junk” volumes
Multiple iterations of heterogeneous
 and homogeneous refinement
Final homogeneous refinement
Non-uniform refinement
3.50Å
PDB: 7ZT6
 EMDB: 14955
93175 particles
SI Figure 1: Single-particle cryo-EM image processing workflow for the Ku70-Ku80-IP6 structure, related to Figures 1 and 2. Schematic representation of particle picking from collected movies using Warp and processing including 2D classification and ab initio reconstruction using cryoSPARC. Upon ab initio reconstructions, multiple rounds of heterogeneous refinement and homogeneous refinement followed. The maps from the initial heterogeneous refinement and final homogeneous refinement are shown, followed by the final non-uniform refinement map with the depicted resolutions for an FSC of 0.143.

## Slide 2
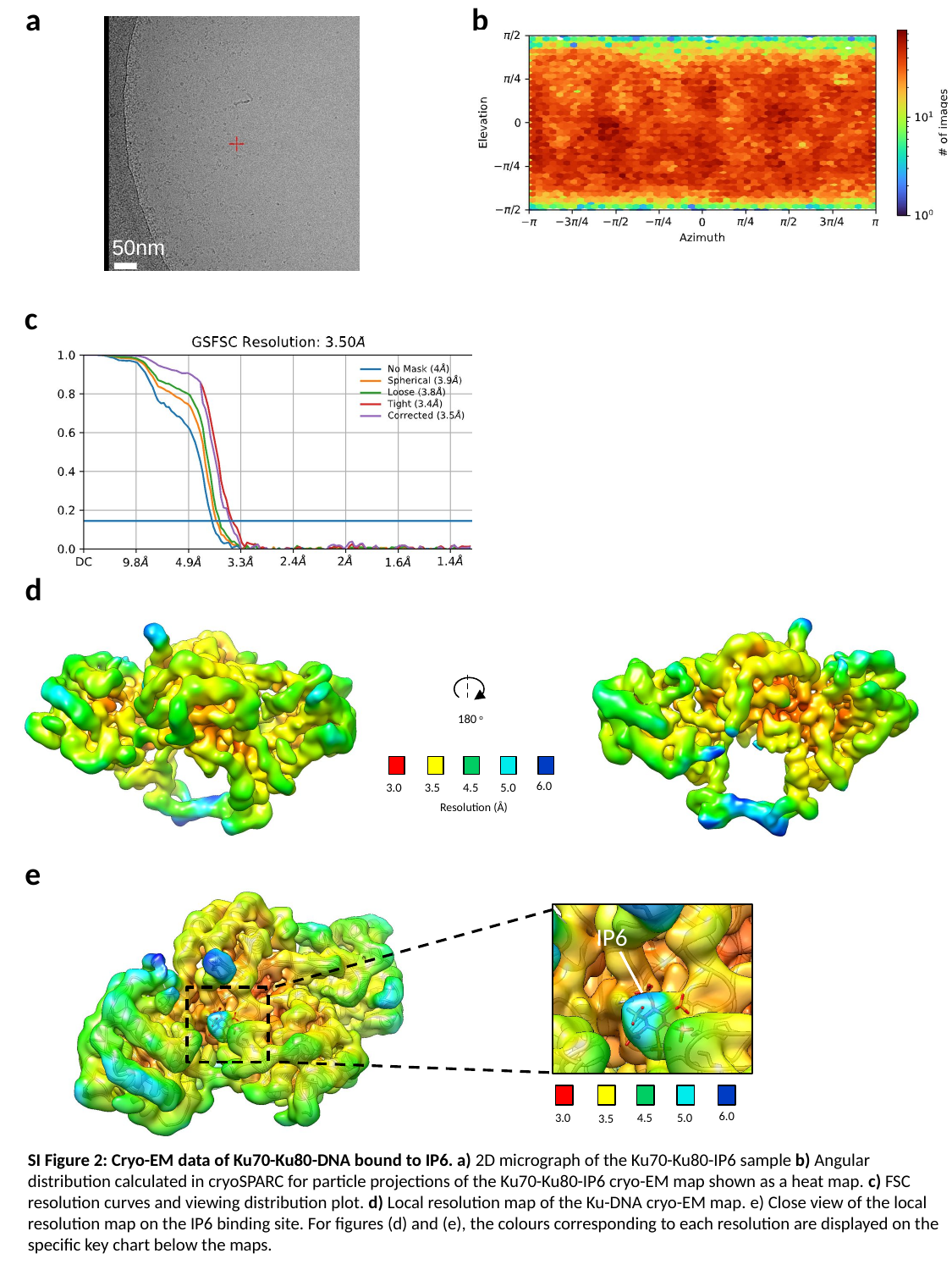

a
b
50nm
c
d
180 o
6.0
5.0
4.5
3.0
3.5
Resolution (Å)
e
IP6
6.0
5.0
4.5
3.0
3.5
SI Figure 2: Cryo-EM data of Ku70-Ku80-DNA bound to IP6. a) 2D micrograph of the Ku70-Ku80-IP6 sample b) Angular distribution calculated in cryoSPARC for particle projections of the Ku70-Ku80-IP6 cryo-EM map shown as a heat map. c) FSC resolution curves and viewing distribution plot. d) Local resolution map of the Ku-DNA cryo-EM map. e) Close view of the local resolution map on the IP6 binding site. For figures (d) and (e), the colours corresponding to each resolution are displayed on the specific key chart below the maps.

## Slide 3
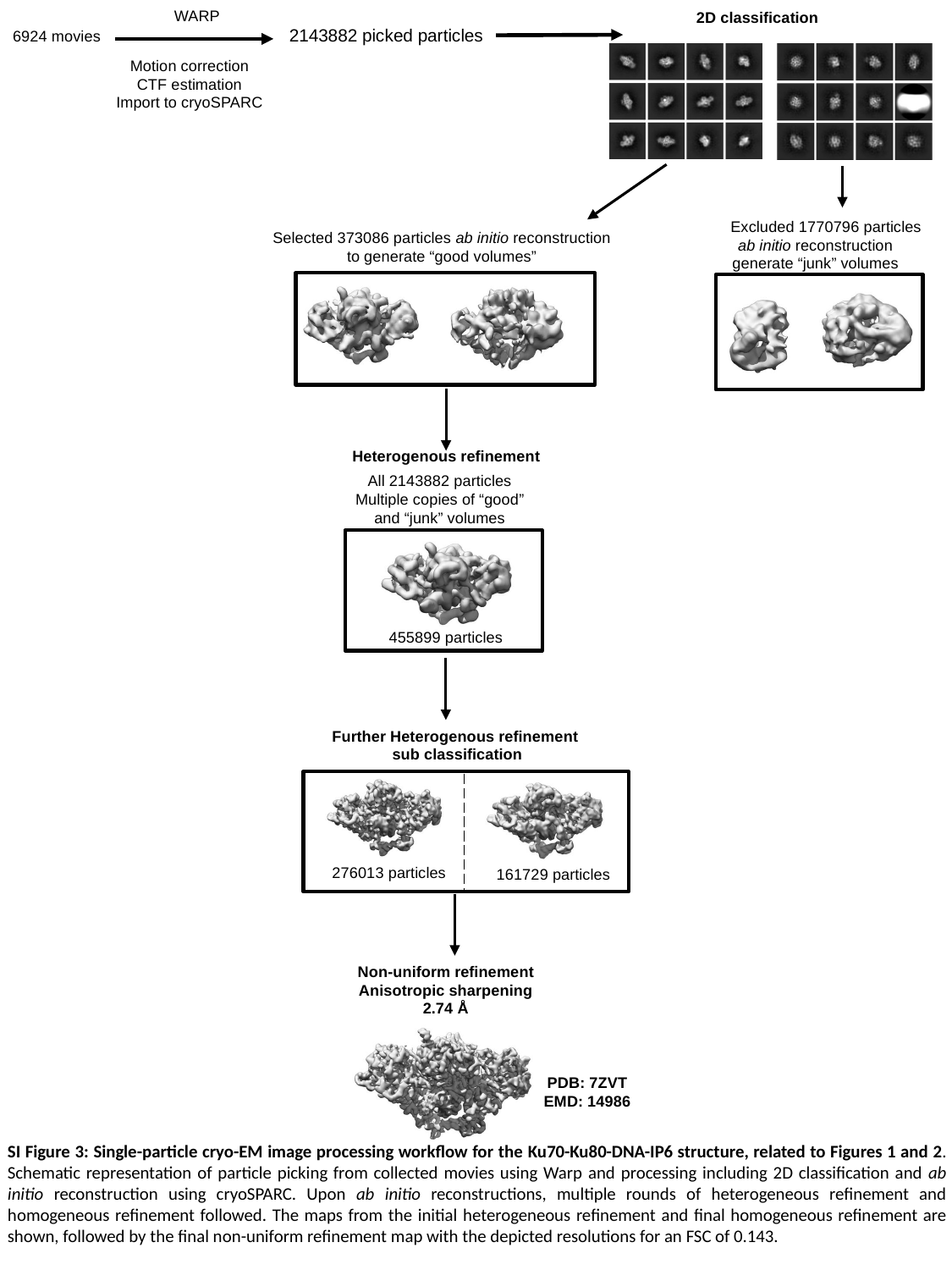

WARP
Motion correction
CTF estimation
Import to cryoSPARC
2D classification
2143882 picked particles
6924 movies
 Excluded 1770796 particles
ab initio reconstruction
generate “junk” volumes
Selected 373086 particles ab initio reconstruction
to generate “good volumes”
Heterogenous refinement
All 2143882 particles
Multiple copies of “good” and “junk” volumes
455899 particles
Further Heterogenous refinement
 sub classification
276013 particles
161729 particles
Non-uniform refinement
Anisotropic sharpening
2.74 Å
PDB: 7ZVT
EMD: 14986
SI Figure 3: Single-particle cryo-EM image processing workflow for the Ku70-Ku80-DNA-IP6 structure, related to Figures 1 and 2. Schematic representation of particle picking from collected movies using Warp and processing including 2D classification and ab initio reconstruction using cryoSPARC. Upon ab initio reconstructions, multiple rounds of heterogeneous refinement and homogeneous refinement followed. The maps from the initial heterogeneous refinement and final homogeneous refinement are shown, followed by the final non-uniform refinement map with the depicted resolutions for an FSC of 0.143.

## Slide 4
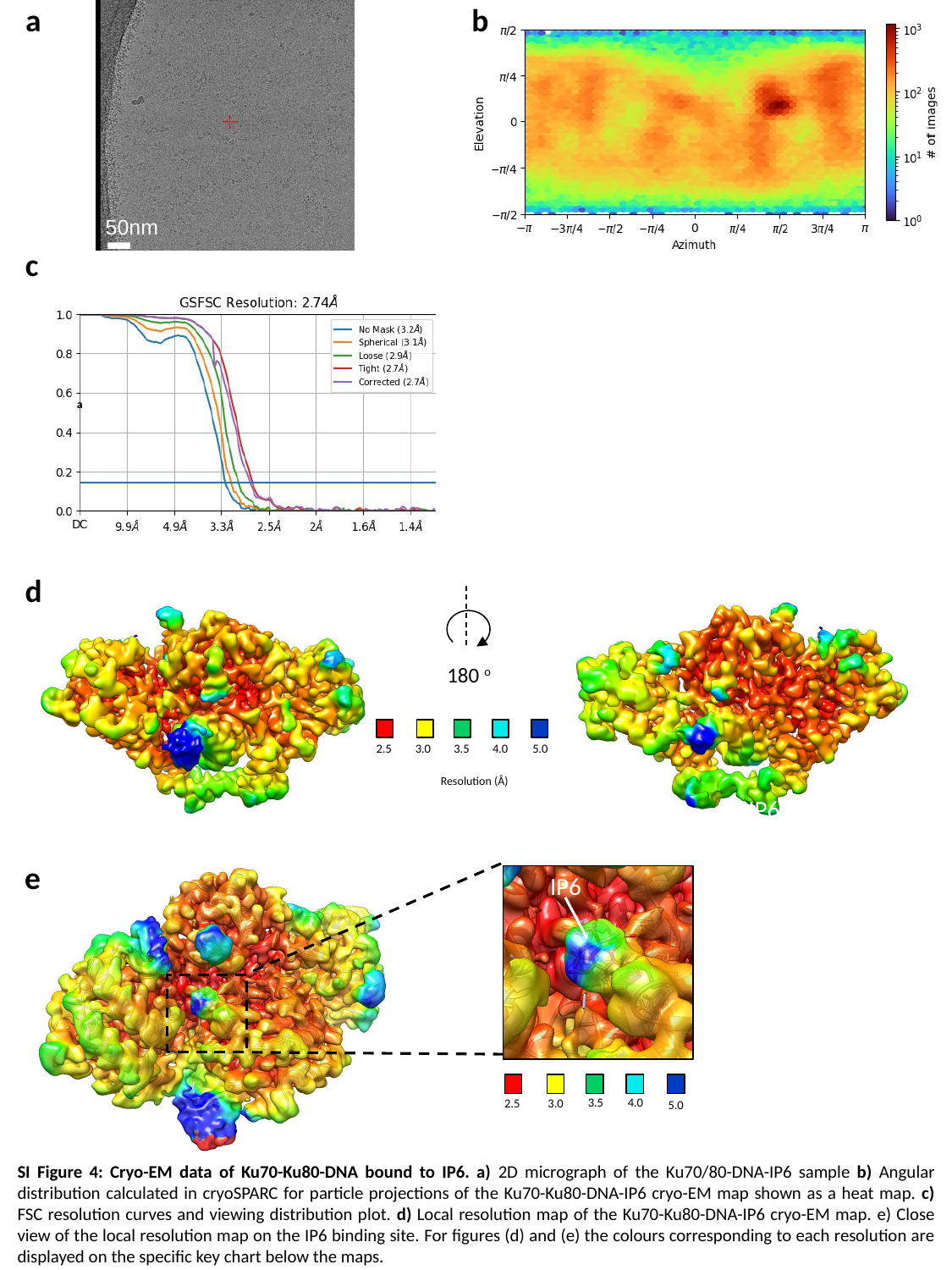

a
b
50nm
c
a
d
180 o
3.0
3.5
4.0
5.0
2.5
Resolution (Å)
IP6
e
IP6
4.0
3.5
2.5
3.0
5.0
SI Figure 4: Cryo-EM data of Ku70-Ku80-DNA bound to IP6. a) 2D micrograph of the Ku70/80-DNA-IP6 sample b) Angular distribution calculated in cryoSPARC for particle projections of the Ku70-Ku80-DNA-IP6 cryo-EM map shown as a heat map. c) FSC resolution curves and viewing distribution plot. d) Local resolution map of the Ku70-Ku80-DNA-IP6 cryo-EM map. e) Close view of the local resolution map on the IP6 binding site. For figures (d) and (e) the colours corresponding to each resolution are displayed on the specific key chart below the maps.

## Slide 5
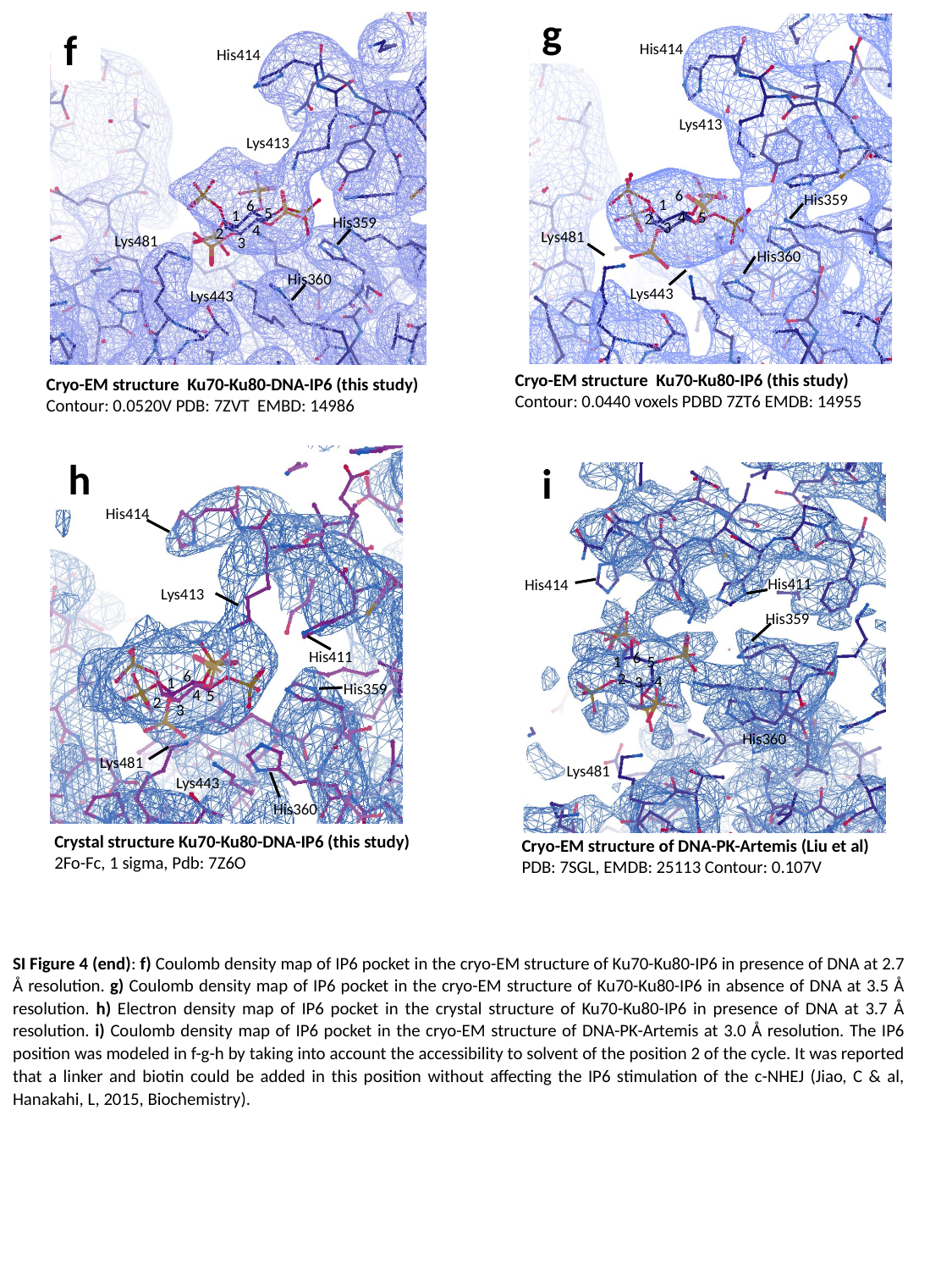

g
f
His414
His414
Lys413
Lys413
6
His359
1
6
5
1
5
4
2
His359
3
4
2
Lys481
Lys481
3
His360
His360
Lys443
Lys443
Cryo-EM structure Ku70-Ku80-IP6 (this study)
Contour: 0.0440 voxels PDBD 7ZT6 EMDB: 14955
Cryo-EM structure Ku70-Ku80-DNA-IP6 (this study)
Contour: 0.0520V PDB: 7ZVT EMBD: 14986
h
i
His414
His411
His414
Lys413
His359
His411
6
1
5
6
2
4
3
1
His359
4
5
2
3
His360
Lys481
Lys481
Lys443
His360
Crystal structure Ku70-Ku80-DNA-IP6 (this study)
2Fo-Fc, 1 sigma, Pdb: 7Z6O
Cryo-EM structure of DNA-PK-Artemis (Liu et al)
PDB: 7SGL, EMDB: 25113 Contour: 0.107V
SI Figure 4 (end): f) Coulomb density map of IP6 pocket in the cryo-EM structure of Ku70-Ku80-IP6 in presence of DNA at 2.7 Å resolution. g) Coulomb density map of IP6 pocket in the cryo-EM structure of Ku70-Ku80-IP6 in absence of DNA at 3.5 Å resolution. h) Electron density map of IP6 pocket in the crystal structure of Ku70-Ku80-IP6 in presence of DNA at 3.7 Å resolution. i) Coulomb density map of IP6 pocket in the cryo-EM structure of DNA-PK-Artemis at 3.0 Å resolution. The IP6 position was modeled in f-g-h by taking into account the accessibility to solvent of the position 2 of the cycle. It was reported that a linker and biotin could be added in this position without affecting the IP6 stimulation of the c-NHEJ (Jiao, C & al, Hanakahi, L, 2015, Biochemistry).

## Slide 6
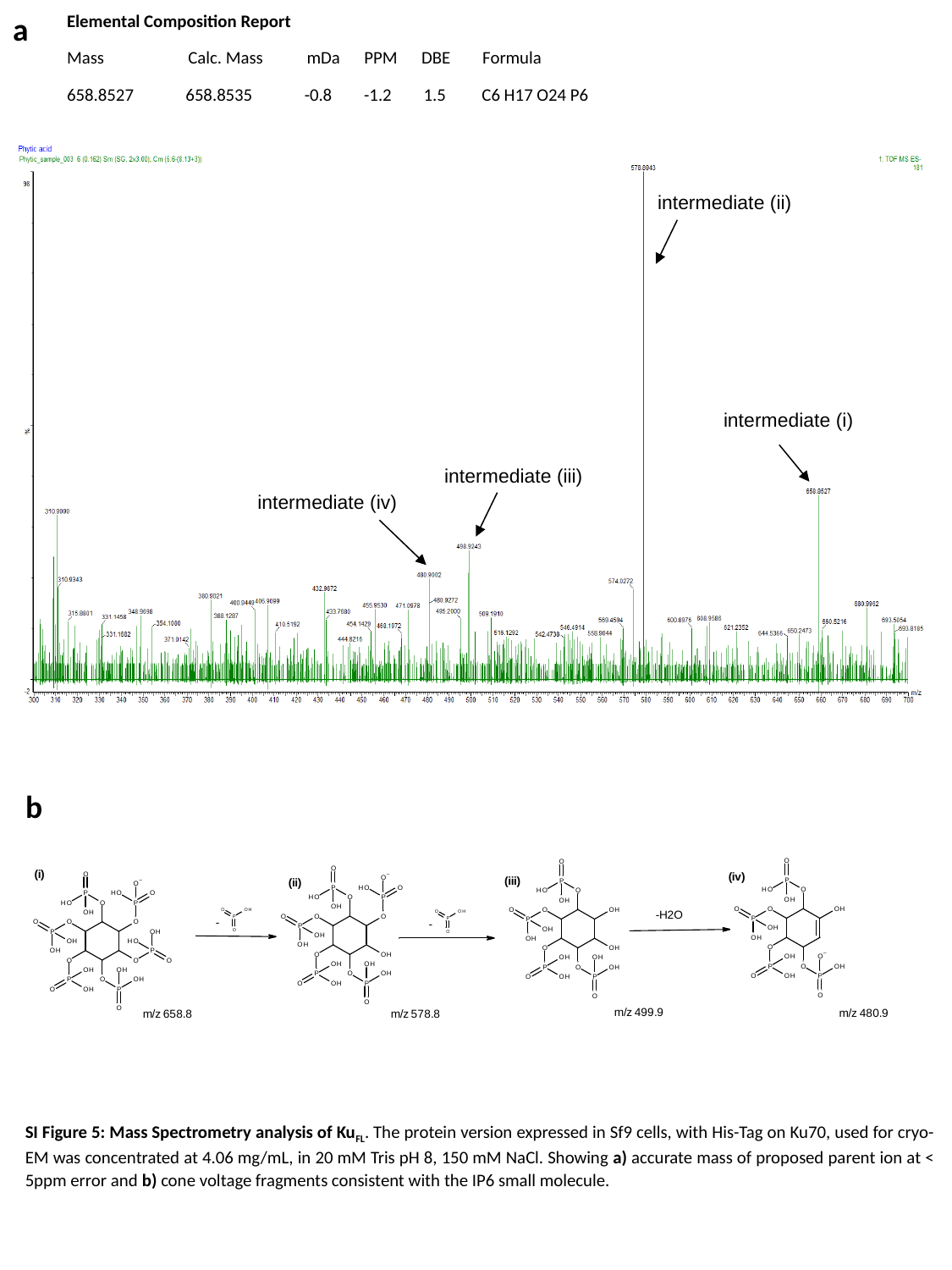

a
Elemental Composition Report
Mass Calc. Mass mDa PPM DBE Formula
658.8527 658.8535 -0.8 -1.2 1.5 C6 H17 O24 P6
intermediate (ii)
intermediate (i)
intermediate (iii)
intermediate (iv)
b
SI Figure 5: Mass Spectrometry analysis of KuFL. The protein version expressed in Sf9 cells, with His-Tag on Ku70, used for cryo-EM was concentrated at 4.06 mg/mL, in 20 mM Tris pH 8, 150 mM NaCl. Showing a) accurate mass of proposed parent ion at < 5ppm error and b) cone voltage fragments consistent with the IP6 small molecule.

## Slide 7
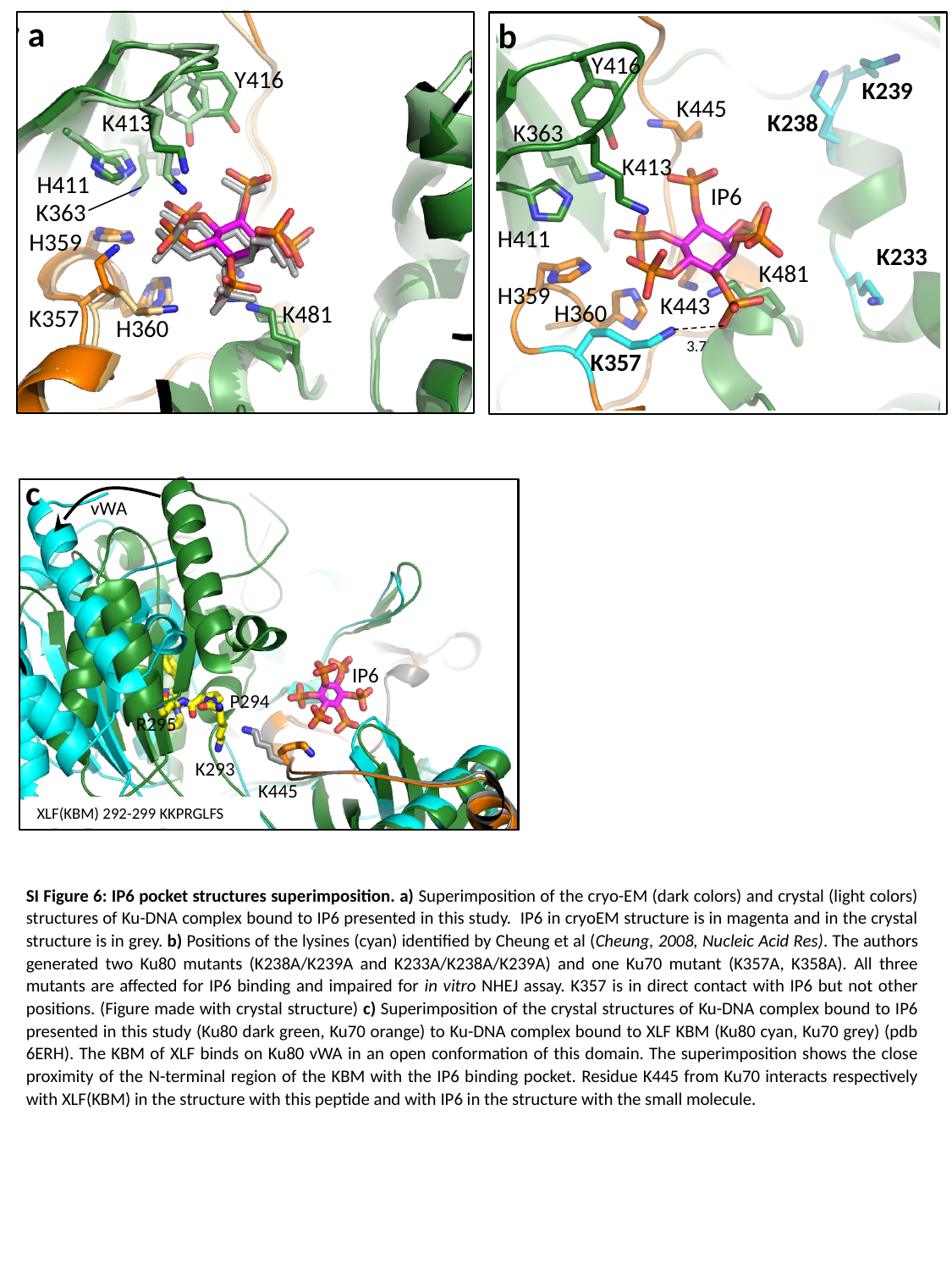

b
a
Y416
Y416
K239
K445
K413
K238
K363
K413
H411
IP6
K363
H411
H359
K233
K481
H359
K443
H360
K481
K357
H360
3.7
K357
c
vWA
IP6
P294
R295
K293
K445
XLF(KBM) 292-299 KKPRGLFS
SI Figure 6: IP6 pocket structures superimposition. a) Superimposition of the cryo-EM (dark colors) and crystal (light colors) structures of Ku-DNA complex bound to IP6 presented in this study. IP6 in cryoEM structure is in magenta and in the crystal structure is in grey. b) Positions of the lysines (cyan) identified by Cheung et al (Cheung, 2008, Nucleic Acid Res). The authors generated two Ku80 mutants (K238A/K239A and K233A/K238A/K239A) and one Ku70 mutant (K357A, K358A). All three mutants are affected for IP6 binding and impaired for in vitro NHEJ assay. K357 is in direct contact with IP6 but not other positions. (Figure made with crystal structure) c) Superimposition of the crystal structures of Ku-DNA complex bound to IP6 presented in this study (Ku80 dark green, Ku70 orange) to Ku-DNA complex bound to XLF KBM (Ku80 cyan, Ku70 grey) (pdb 6ERH). The KBM of XLF binds on Ku80 vWA in an open conformation of this domain. The superimposition shows the close proximity of the N-terminal region of the KBM with the IP6 binding pocket. Residue K445 from Ku70 interacts respectively with XLF(KBM) in the structure with this peptide and with IP6 in the structure with the small molecule.

## Slide 8
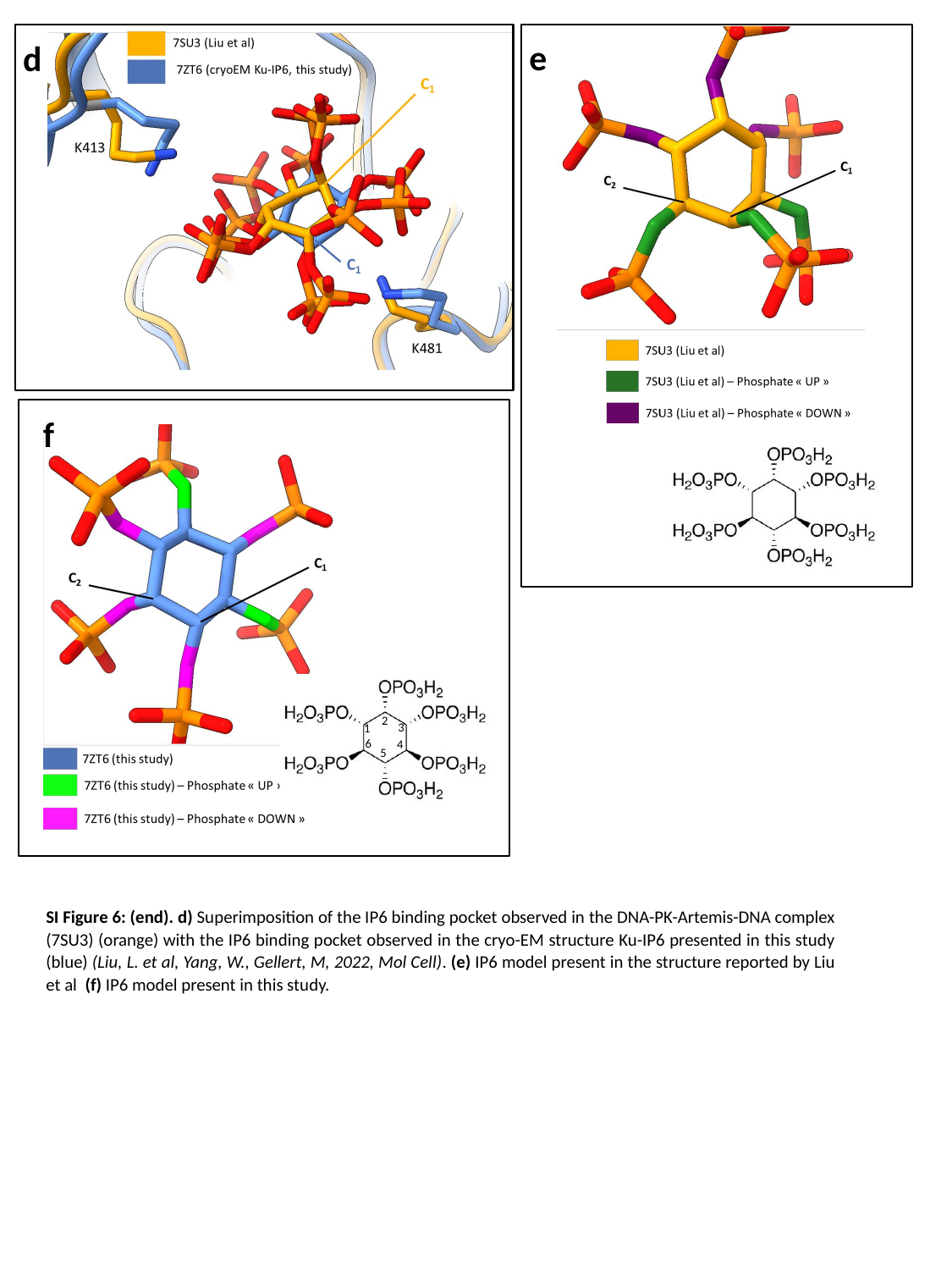

e
d
f
2
3
1
6
4
5
SI Figure 6: (end). d) Superimposition of the IP6 binding pocket observed in the DNA-PK-Artemis-DNA complex (7SU3) (orange) with the IP6 binding pocket observed in the cryo-EM structure Ku-IP6 presented in this study (blue) (Liu, L. et al, Yang, W., Gellert, M, 2022, Mol Cell). (e) IP6 model present in the structure reported by Liu et al (f) IP6 model present in this study.

## Slide 9
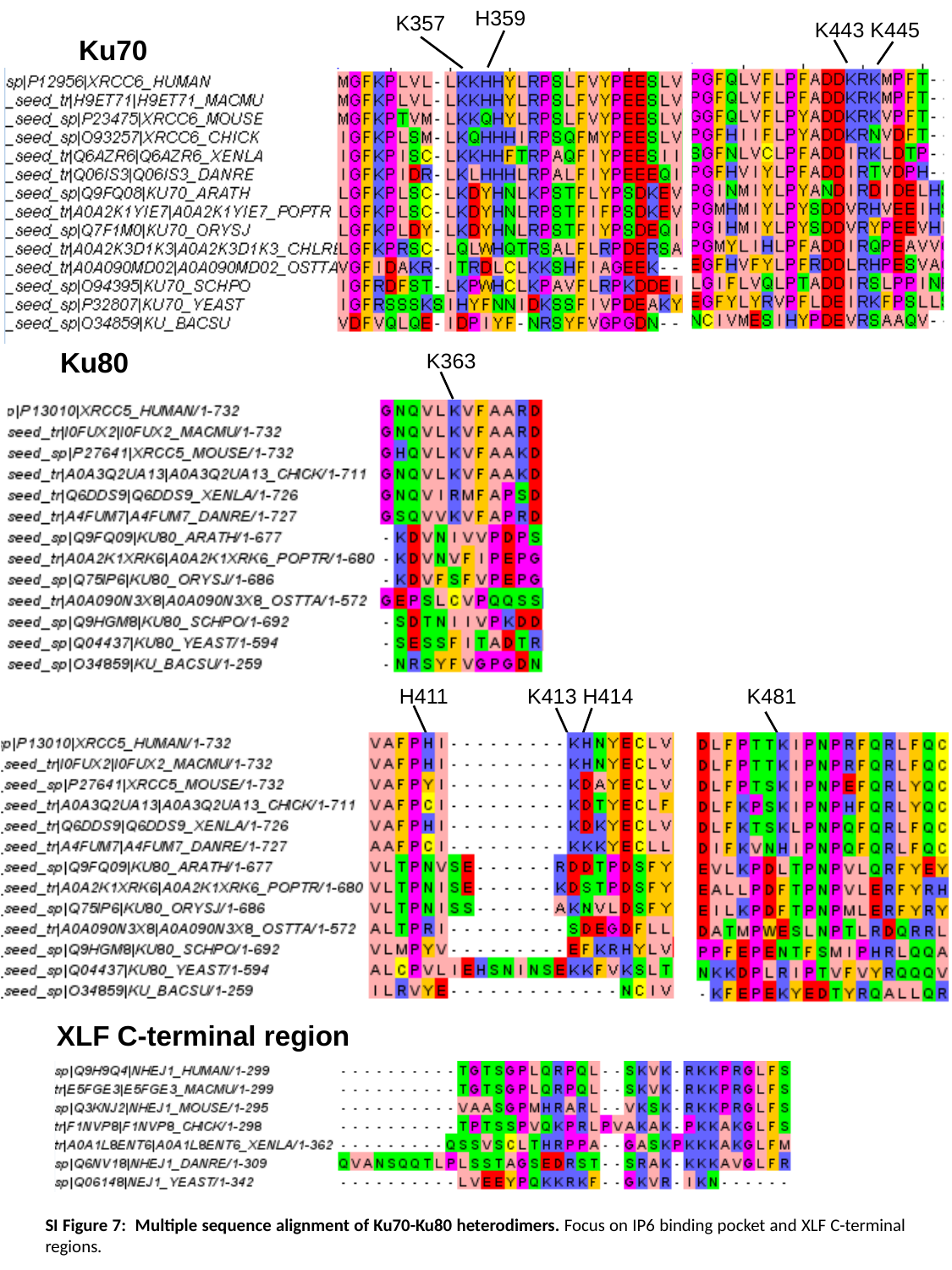

H359
K357
K443 K445
Ku70
Ku80
K363
H411
K413 H414
K481
XLF C-terminal region
SI Figure 7: Multiple sequence alignment of Ku70-Ku80 heterodimers. Focus on IP6 binding pocket and XLF C-terminal regions.

## Slide 10
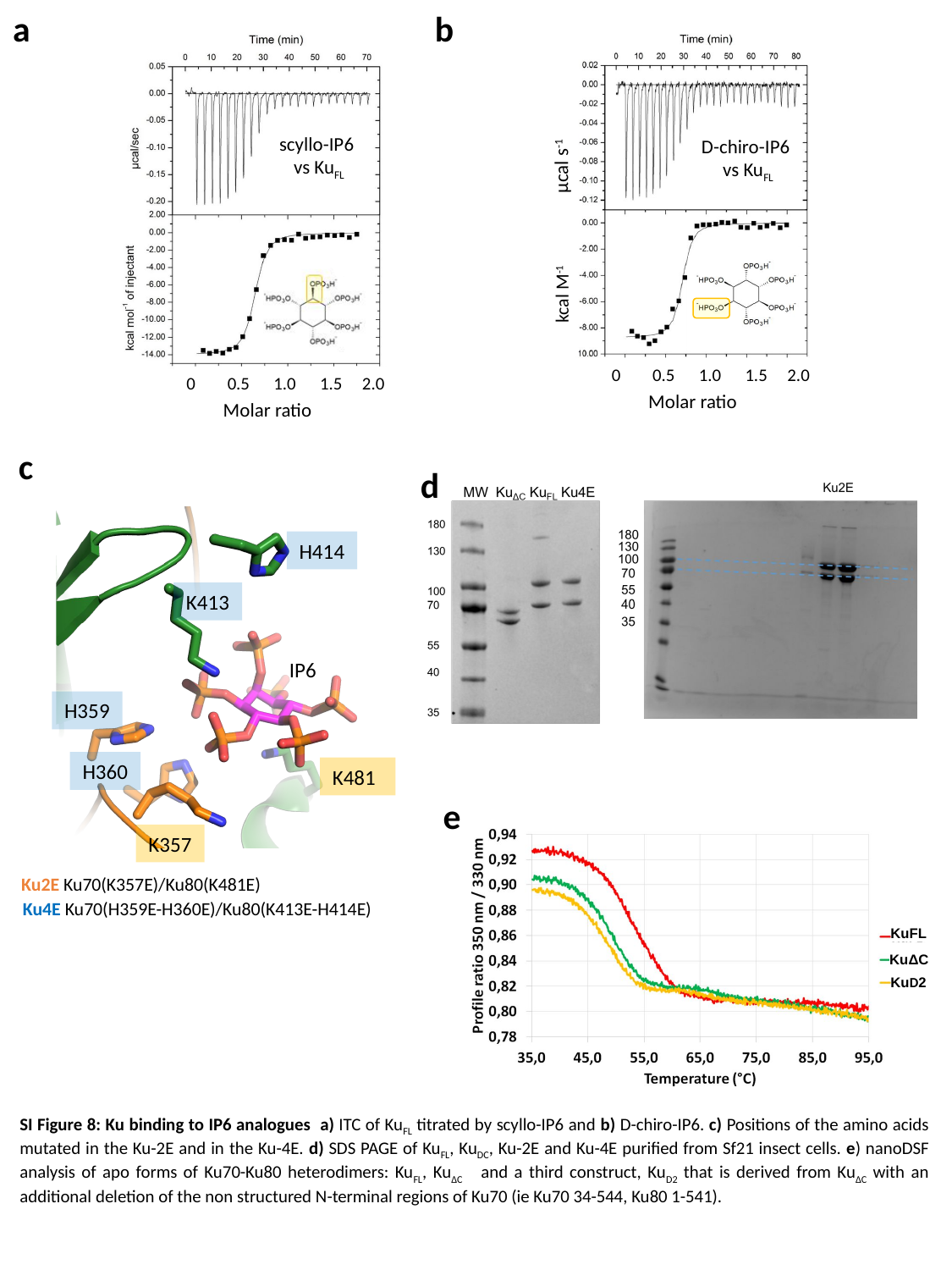

a
b
µcal s-1
scyllo-IP6
 vs KuFL
D-chiro-IP6
 vs KuFL
kcal M-1
0 0.5 1.0 1.5 2.0
0 0.5 1.0 1.5 2.0
Molar ratio
Molar ratio
c
d
H414
K413
IP6
H359
H360
K481
K357
Ku2E Ku70(K357E)/Ku80(K481E)
Ku4E Ku70(H359E-H360E)/Ku80(K413E-H414E)
e
KuFL
KuΔC
KuD2
SI Figure 8: Ku binding to IP6 analogues a) ITC of KuFL titrated by scyllo-IP6 and b) D-chiro-IP6. c) Positions of the amino acids mutated in the Ku-2E and in the Ku-4E. d) SDS PAGE of KuFL, KuDC, Ku-2E and Ku-4E purified from Sf21 insect cells. e) nanoDSF analysis of apo forms of Ku70-Ku80 heterodimers: KuFL, KuΔC and a third construct, KuD2 that is derived from KuΔC with an additional deletion of the non structured N-terminal regions of Ku70 (ie Ku70 34-544, Ku80 1-541).

## Slide 11
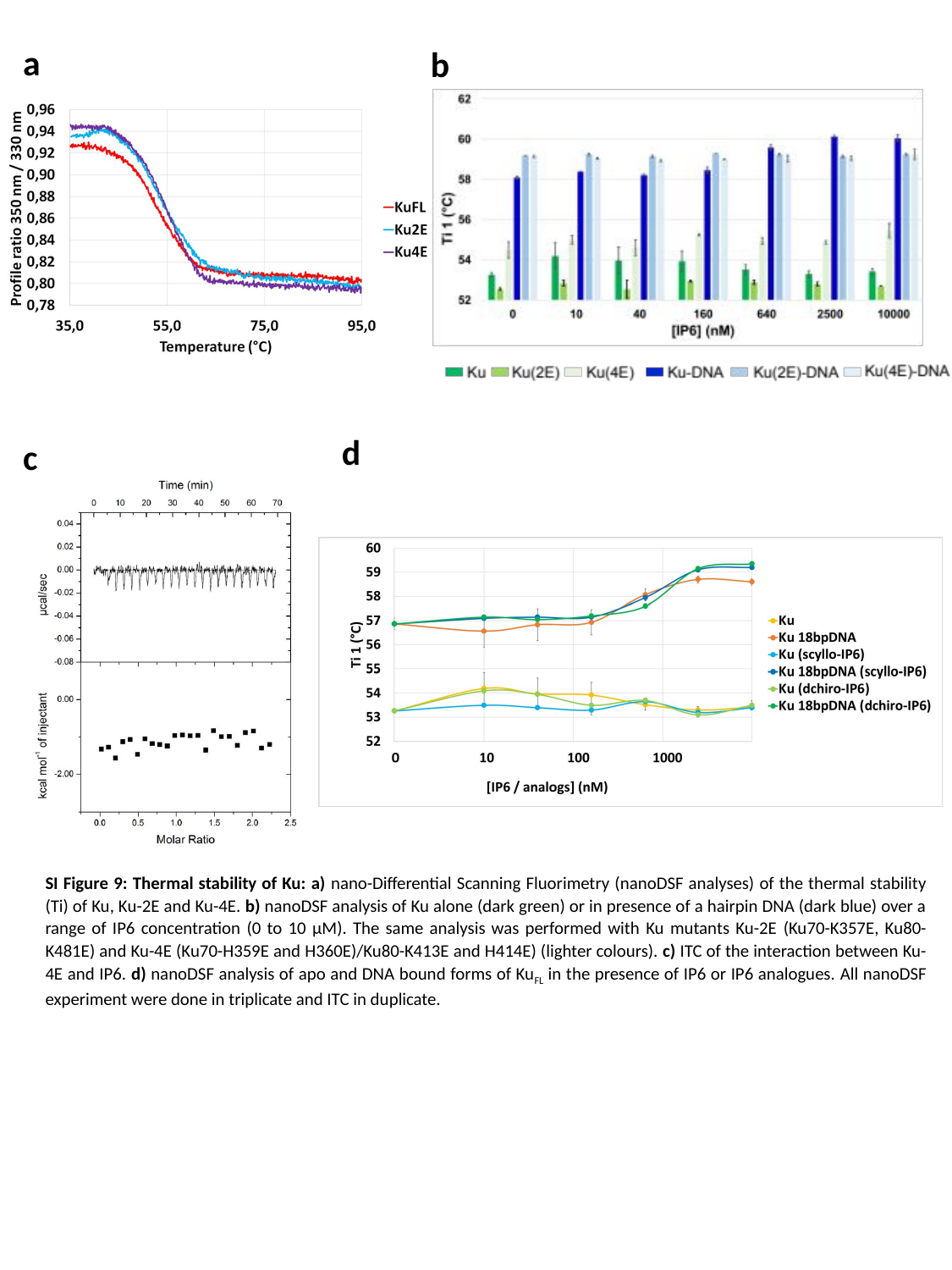

a
b
d
c
SI Figure 9: Thermal stability of Ku: a) nano-Differential Scanning Fluorimetry (nanoDSF analyses) of the thermal stability (Ti) of Ku, Ku-2E and Ku-4E. b) nanoDSF analysis of Ku alone (dark green) or in presence of a hairpin DNA (dark blue) over a range of IP6 concentration (0 to 10 µM). The same analysis was performed with Ku mutants Ku-2E (Ku70-K357E, Ku80-K481E) and Ku-4E (Ku70-H359E and H360E)/Ku80-K413E and H414E) (lighter colours). c) ITC of the interaction between Ku-4E and IP6. d) nanoDSF analysis of apo and DNA bound forms of KuFL in the presence of IP6 or IP6 analogues. All nanoDSF experiment were done in triplicate and ITC in duplicate.

## Slide 12
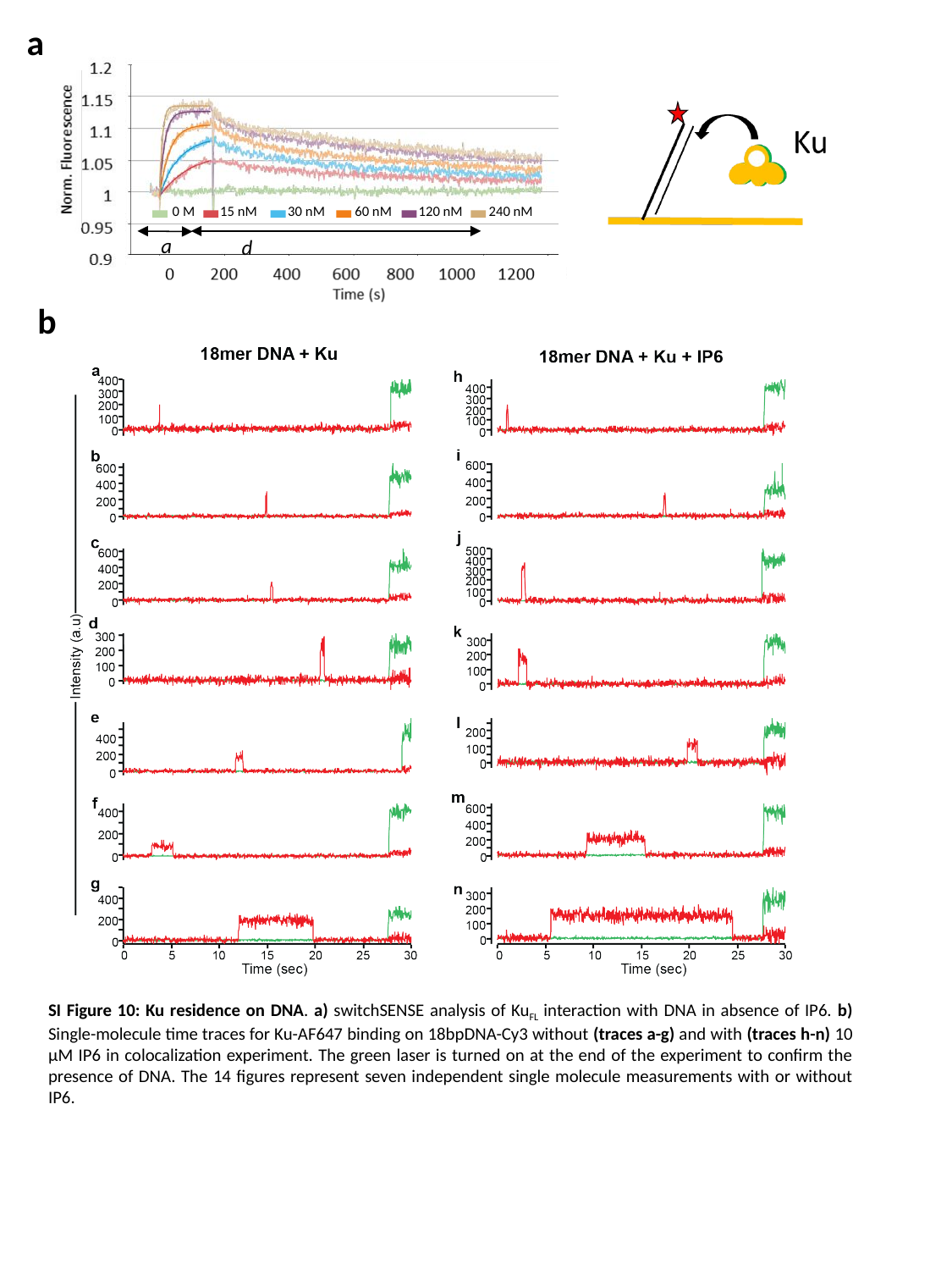

a
0 M
15 nM
30 nM
60 nM
120 nM
240 nM
a
d
b
SI Figure 10: Ku residence on DNA. a) switchSENSE analysis of KuFL interaction with DNA in absence of IP6. b) Single-molecule time traces for Ku-AF647 binding on 18bpDNA-Cy3 without (traces a-g) and with (traces h-n) 10 µM IP6 in colocalization experiment. The green laser is turned on at the end of the experiment to confirm the presence of DNA. The 14 figures represent seven independent single molecule measurements with or without IP6.

## Slide 13
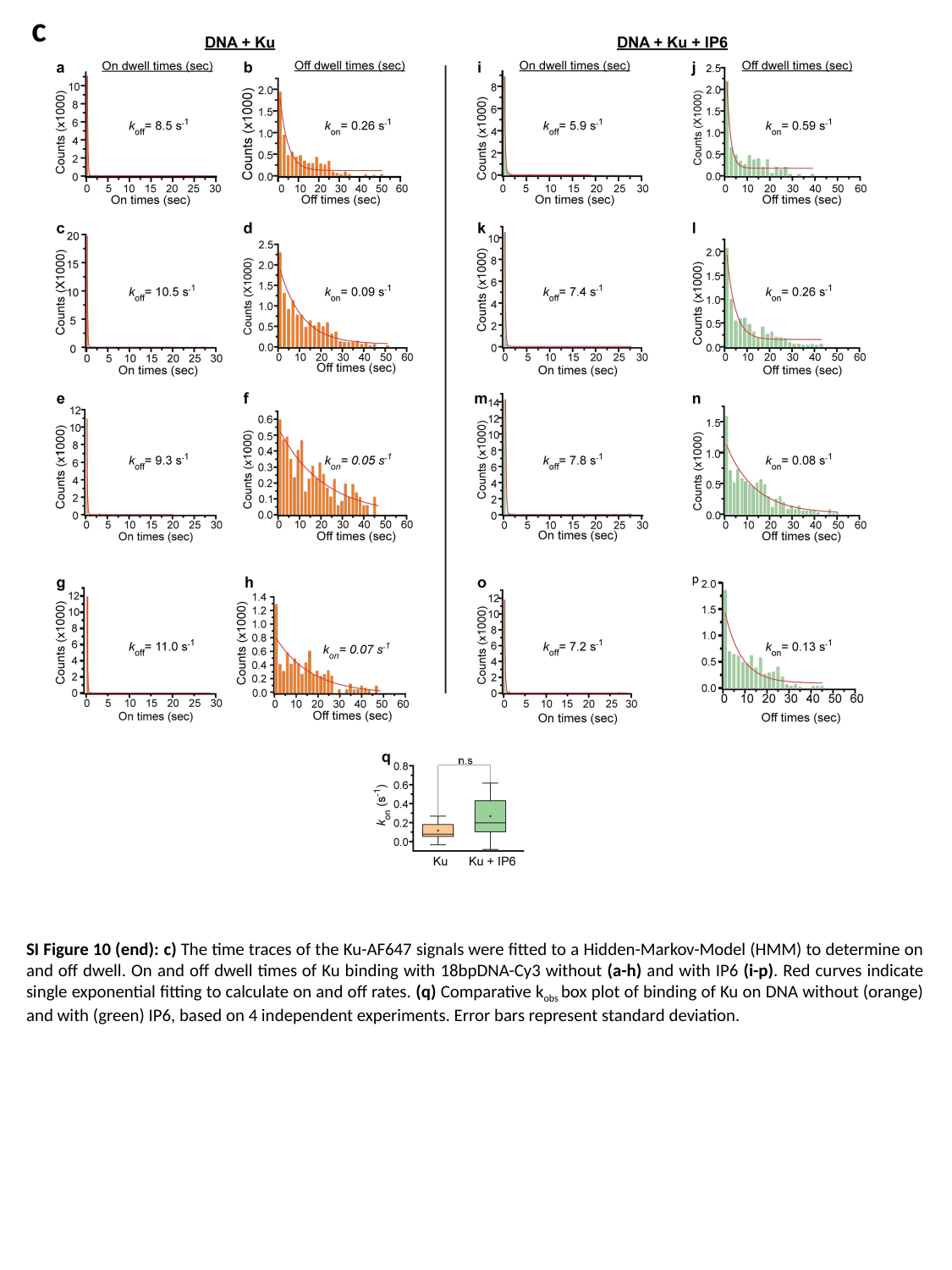

c
SI Figure 10 (end): c) The time traces of the Ku-AF647 signals were fitted to a Hidden-Markov-Model (HMM) to determine on and off dwell. On and off dwell times of Ku binding with 18bpDNA-Cy3 without (a-h) and with IP6 (i-p). Red curves indicate single exponential fitting to calculate on and off rates. (q) Comparative kobs box plot of binding of Ku on DNA without (orange) and with (green) IP6, based on 4 independent experiments. Error bars represent standard deviation.

## Slide 14
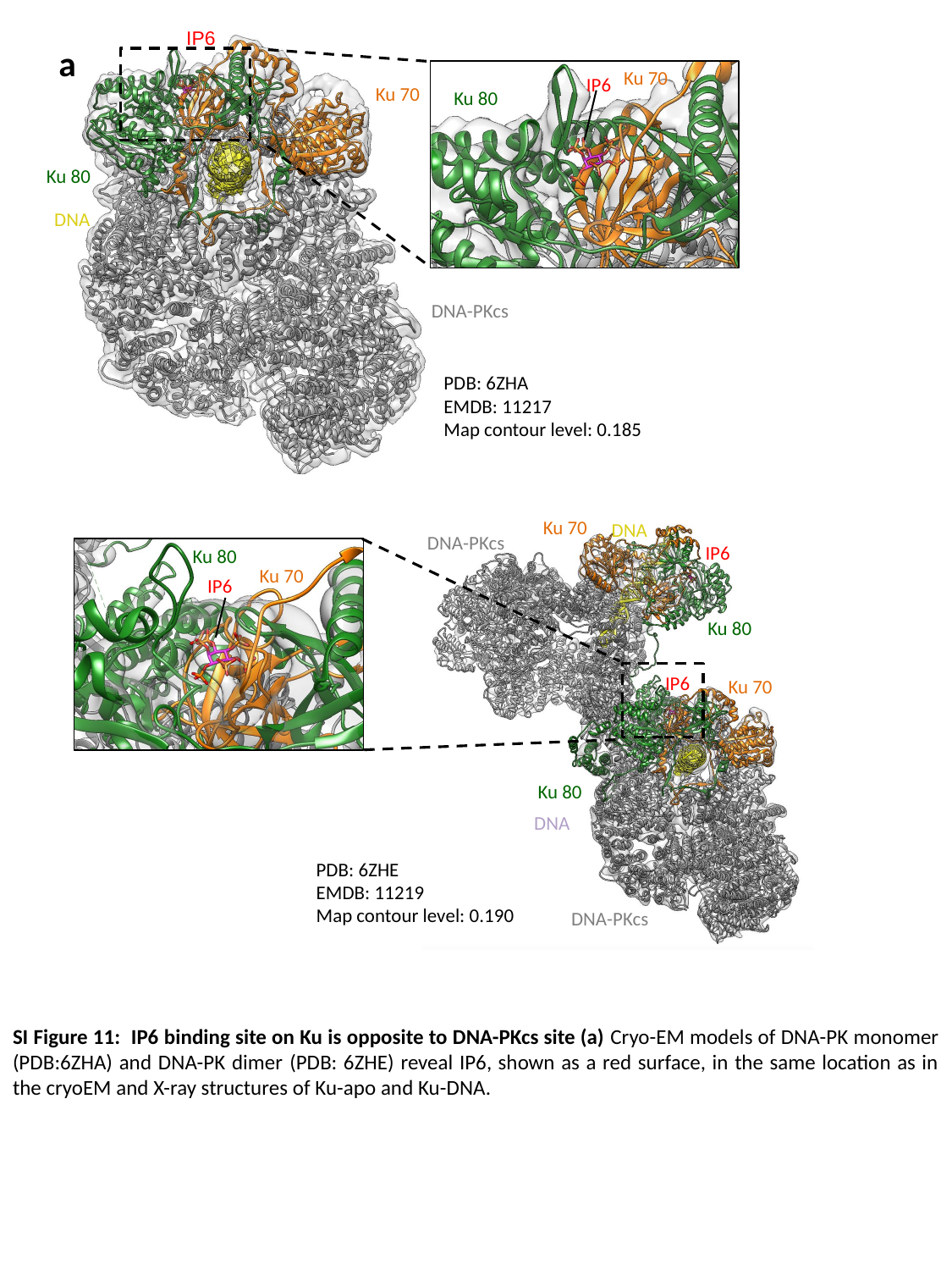

IP6
a
Ku 70
IP6
Ku 70
Ku 80
Ku 80
DNA
DNA-PKcs
PDB: 6ZHA
EMDB: 11217
Map contour level: 0.185
Ku 70
DNA
DNA-PKcs
IP6
Ku 80
Ku 70
IP6
DNA-PKcs
Ku 80
IP6
Ku 70
Ku 80
DNA
PDB: 6ZHE
EMDB: 11219
Map contour level: 0.190
DNA-PKcs
SI Figure 11: IP6 binding site on Ku is opposite to DNA-PKcs site (a) Cryo-EM models of DNA-PK monomer (PDB:6ZHA) and DNA-PK dimer (PDB: 6ZHE) reveal IP6, shown as a red surface, in the same location as in the cryoEM and X-ray structures of Ku-apo and Ku-DNA.

## Slide 15
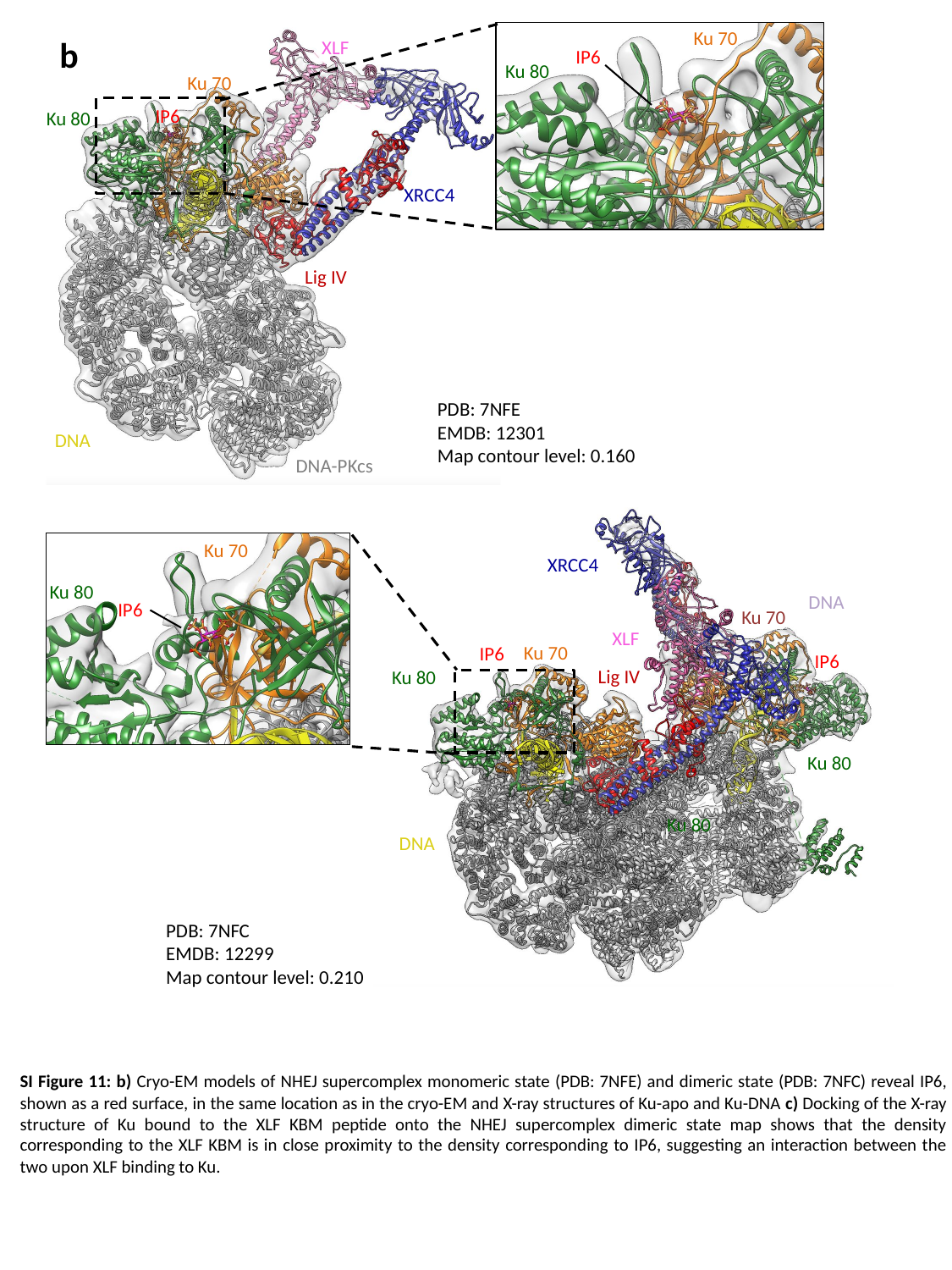

Ku 70
b
XLF
IP6
Ku 80
Ku 70
IP6
Ku 80
XRCC4
Lig IV
PDB: 7NFE
EMDB: 12301
Map contour level: 0.160
DNA
DNA-PKcs
Ku 70
XRCC4
Ku 80
DNA
IP6
Ku 70
XLF
Ku 70
IP6
IP6
Lig IV
Ku 80
Ku 80
Ku 80
DNA
PDB: 7NFC
EMDB: 12299
Map contour level: 0.210
SI Figure 11: b) Cryo-EM models of NHEJ supercomplex monomeric state (PDB: 7NFE) and dimeric state (PDB: 7NFC) reveal IP6, shown as a red surface, in the same location as in the cryo-EM and X-ray structures of Ku-apo and Ku-DNA c) Docking of the X-ray structure of Ku bound to the XLF KBM peptide onto the NHEJ supercomplex dimeric state map shows that the density corresponding to the XLF KBM is in close proximity to the density corresponding to IP6, suggesting an interaction between the two upon XLF binding to Ku.

## Slide 16
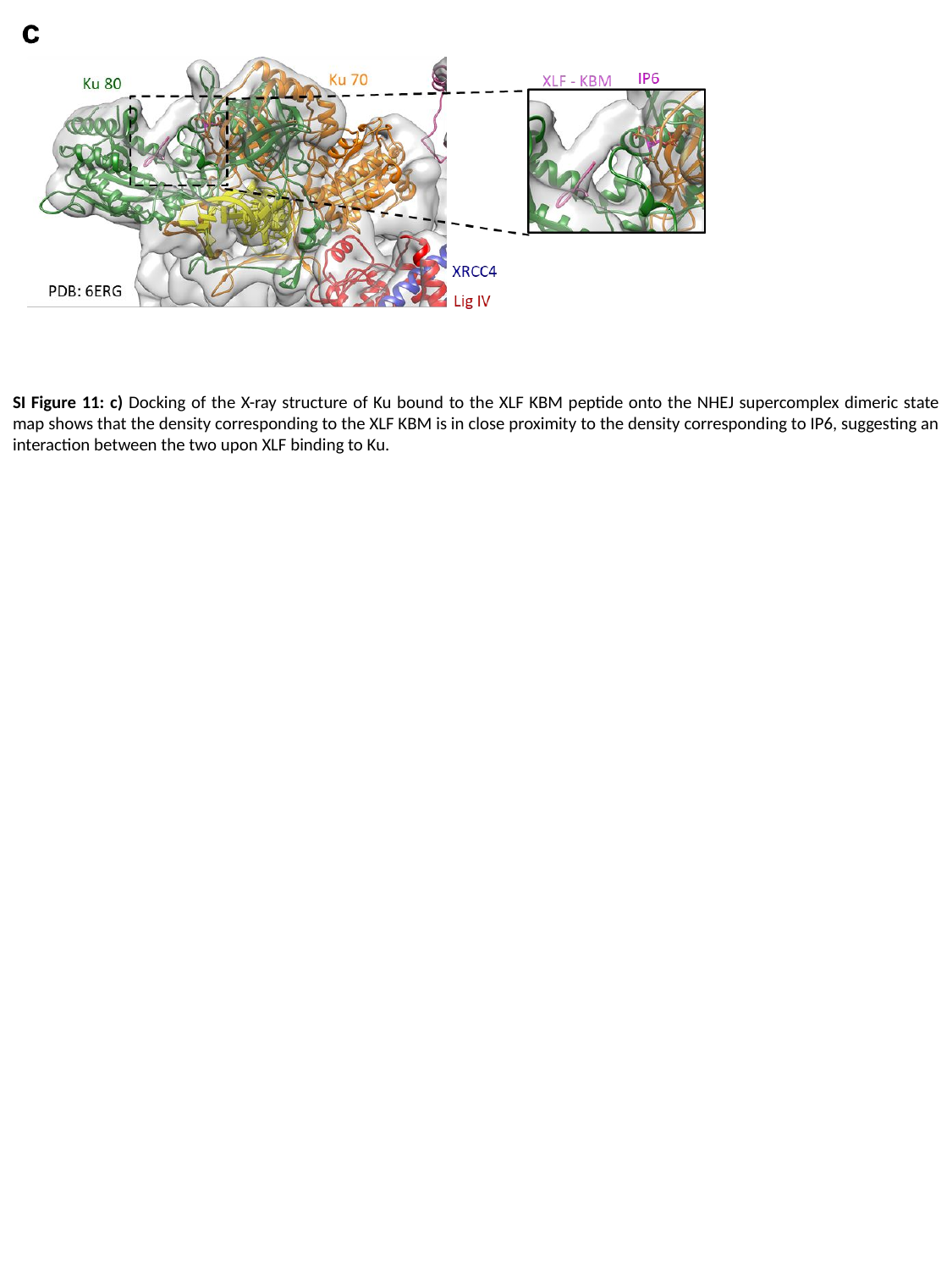

SI Figure 11: c) Docking of the X-ray structure of Ku bound to the XLF KBM peptide onto the NHEJ supercomplex dimeric state map shows that the density corresponding to the XLF KBM is in close proximity to the density corresponding to IP6, suggesting an interaction between the two upon XLF binding to Ku.

## Slide 17
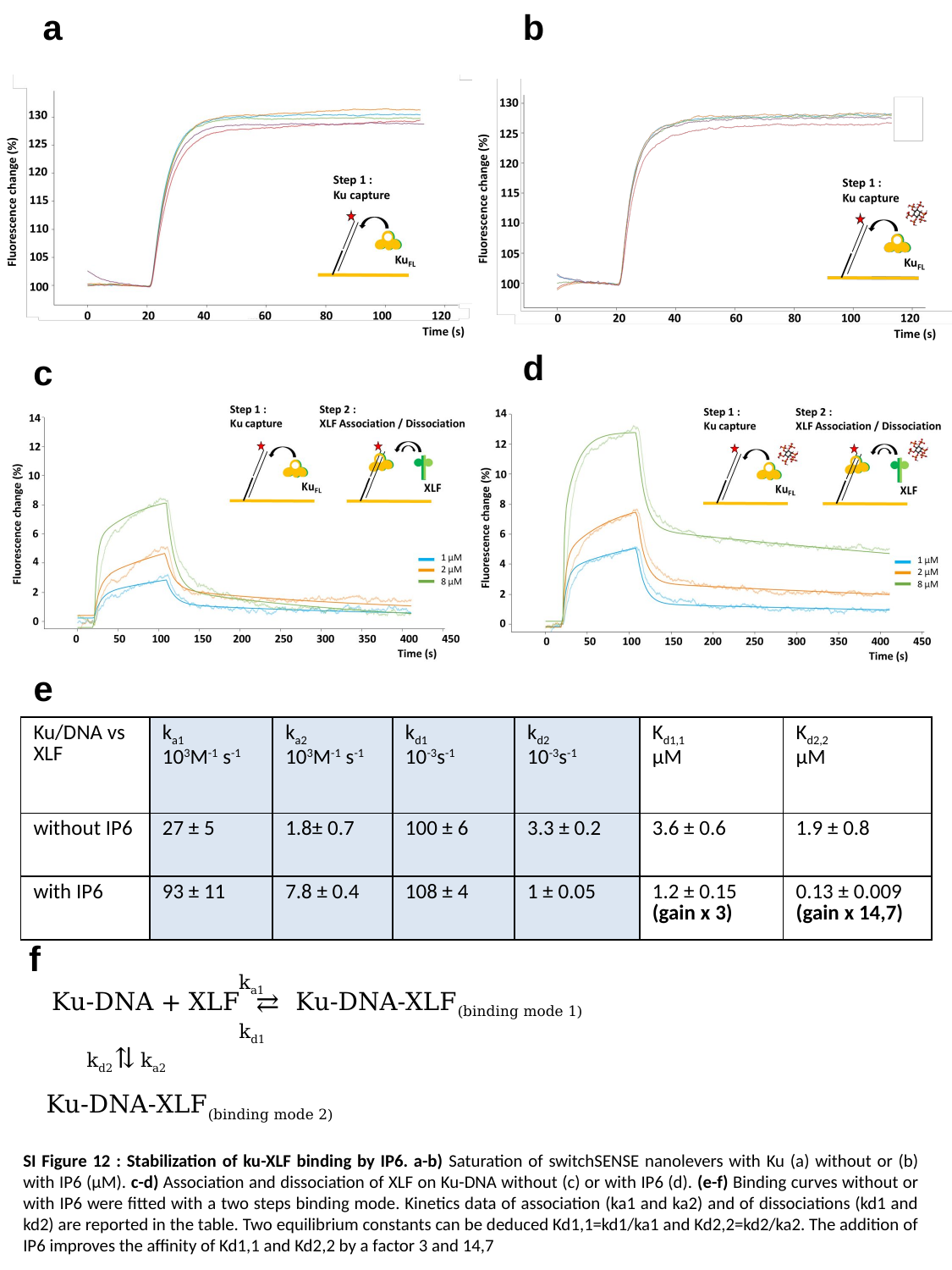

a
b
d
c
e
| Ku/DNA vs XLF | ka1 103M-1 s-1 | ka2 103M-1 s-1 | kd1 10-3s-1 | kd2 10-3s-1 | Kd1,1 µM | Kd2,2 µM |
| --- | --- | --- | --- | --- | --- | --- |
| without IP6 | 27 ± 5 | 1.8± 0.7 | 100 ± 6 | 3.3 ± 0.2 | 3.6 ± 0.6 | 1.9 ± 0.8 |
| with IP6 | 93 ± 11 | 7.8 ± 0.4 | 108 ± 4 | 1 ± 0.05 | 1.2 ± 0.15 (gain x 3) | 0.13 ± 0.009 (gain x 14,7) |
ka1
Ku-DNA + XLF ⇄ Ku-DNA-XLF(binding mode 1)
kd1
⇅
kd2
ka2
Ku-DNA-XLF(binding mode 2)
f
SI Figure 12 : Stabilization of ku-XLF binding by IP6. a-b) Saturation of switchSENSE nanolevers with Ku (a) without or (b) with IP6 (µM). c-d) Association and dissociation of XLF on Ku-DNA without (c) or with IP6 (d). (e-f) Binding curves without or with IP6 were fitted with a two steps binding mode. Kinetics data of association (ka1 and ka2) and of dissociations (kd1 and kd2) are reported in the table. Two equilibrium constants can be deduced Kd1,1=kd1/ka1 and Kd2,2=kd2/ka2. The addition of IP6 improves the affinity of Kd1,1 and Kd2,2 by a factor 3 and 14,7

## Slide 18
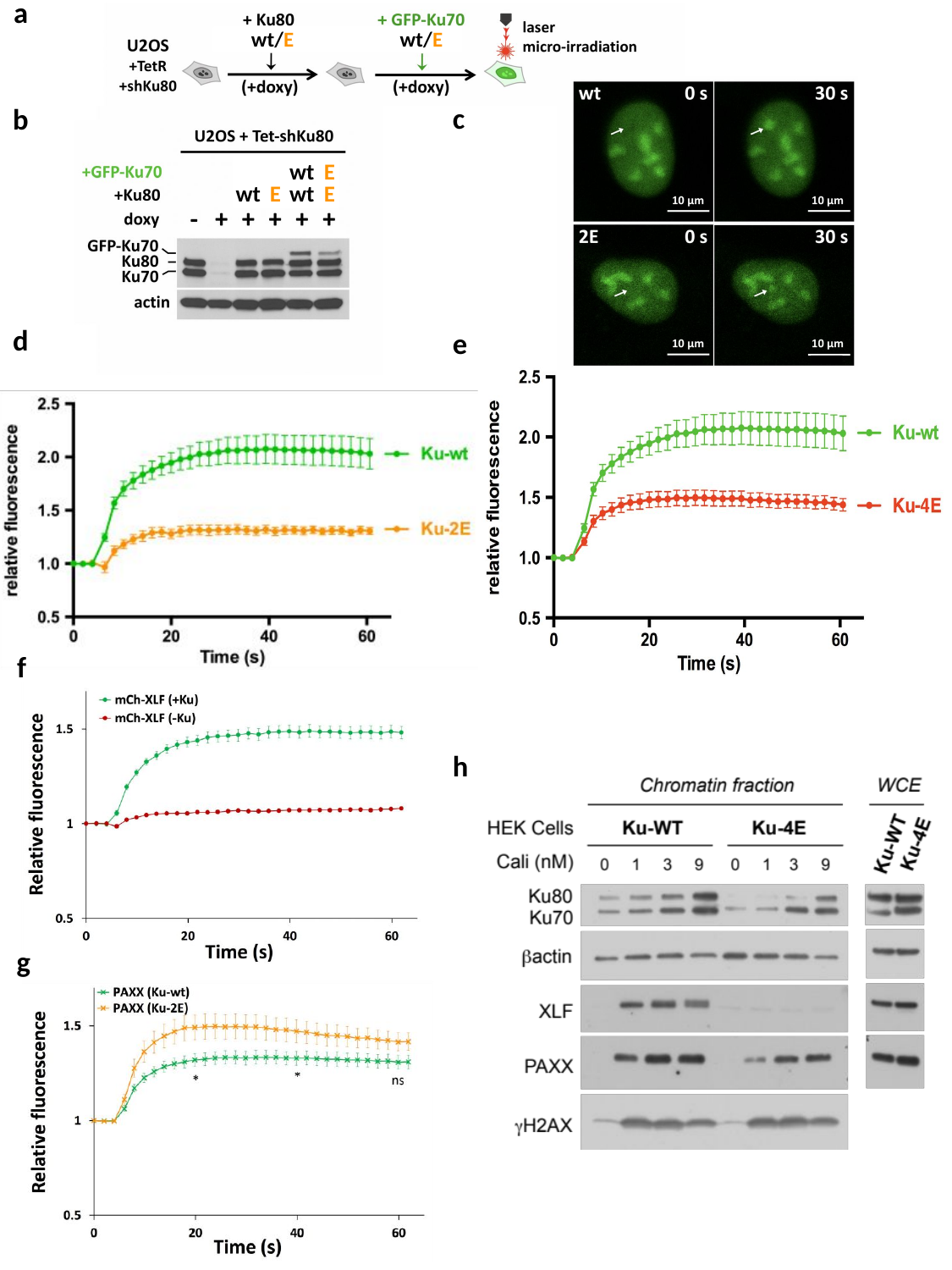

a
wt/E
wt/E
wt
E
wt
E
wt
E
c
b
d
e
f
h
g

## Slide 19
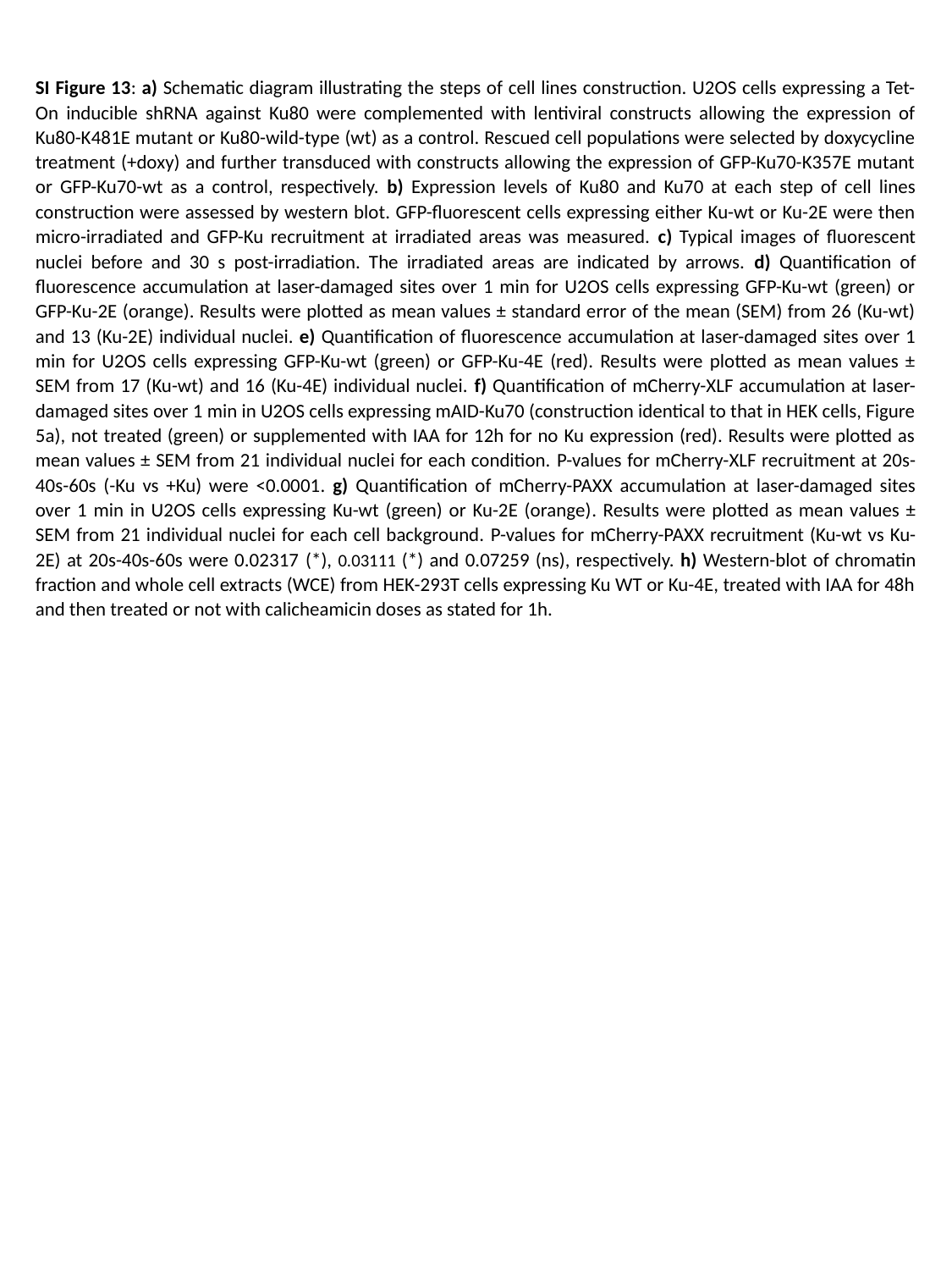

SI Figure 13: a) Schematic diagram illustrating the steps of cell lines construction. U2OS cells expressing a Tet-On inducible shRNA against Ku80 were complemented with lentiviral constructs allowing the expression of Ku80-K481E mutant or Ku80-wild-type (wt) as a control. Rescued cell populations were selected by doxycycline treatment (+doxy) and further transduced with constructs allowing the expression of GFP-Ku70-K357E mutant or GFP-Ku70-wt as a control, respectively. b) Expression levels of Ku80 and Ku70 at each step of cell lines construction were assessed by western blot. GFP-fluorescent cells expressing either Ku-wt or Ku-2E were then micro-irradiated and GFP-Ku recruitment at irradiated areas was measured. c) Typical images of fluorescent nuclei before and 30 s post-irradiation. The irradiated areas are indicated by arrows. d) Quantification of fluorescence accumulation at laser-damaged sites over 1 min for U2OS cells expressing GFP-Ku-wt (green) or GFP-Ku-2E (orange). Results were plotted as mean values ± standard error of the mean (SEM) from 26 (Ku-wt) and 13 (Ku-2E) individual nuclei. e) Quantification of fluorescence accumulation at laser-damaged sites over 1 min for U2OS cells expressing GFP-Ku-wt (green) or GFP-Ku-4E (red). Results were plotted as mean values ± SEM from 17 (Ku-wt) and 16 (Ku-4E) individual nuclei. f) Quantification of mCherry-XLF accumulation at laser-damaged sites over 1 min in U2OS cells expressing mAID-Ku70 (construction identical to that in HEK cells, Figure 5a), not treated (green) or supplemented with IAA for 12h for no Ku expression (red). Results were plotted as mean values ± SEM from 21 individual nuclei for each condition. P-values for mCherry-XLF recruitment at 20s-40s-60s (-Ku vs +Ku) were <0.0001. g) Quantification of mCherry-PAXX accumulation at laser-damaged sites over 1 min in U2OS cells expressing Ku-wt (green) or Ku-2E (orange). Results were plotted as mean values ± SEM from 21 individual nuclei for each cell background. P-values for mCherry-PAXX recruitment (Ku-wt vs Ku-2E) at 20s-40s-60s were 0.02317 (*), 0.03111 (*) and 0.07259 (ns), respectively. h) Western-blot of chromatin fraction and whole cell extracts (WCE) from HEK-293T cells expressing Ku WT or Ku-4E, treated with IAA for 48h and then treated or not with calicheamicin doses as stated for 1h.
