# Supplementary material for: Structural and functional basis of inositol hexaphosphate stimulation of NHEJ through stabilization of Ku-XLF interaction
Source: Nucleic Acids Res. 2023 Oct 23;51(21):11732–47. doi: 10.1093/nar/gkad863 (PMC10682503; doi:10.1093/nar/gkad863)
Supplement: gkad863_Supplemental_Files [file gkad863_supplemental_files.zip › Ku-IP6 SI Material and Methods v17 clean.docx]

## SUPPLEMENTARY INFORMATION - A Kefala Stavridi, et al

## Material and Methods

## Reagents IP6. Phytic acid sodium salt hydrate (Sigma Aldrich P8810), D-chiro-inositol 1,2,3,4,5,6-hexakisphosphate sodium salt (Bertin BIOREAGENT 9002341), scyllo-inositol hexakisphosphate sodium salt (Bertin Bioreagent 9002339)

## Oligonucleotides. For cryo-EM experiments, oligonucleotides were synthesized as single strands oligonucleotides (Sigma Aldrich). Oligonucleotides were synthesized and purified by HPLC. Each oligonucleotides was prepared as a 100 µM stock. The oligo complementary pairs were mixed at a 1:1 ratio to a final concentration of 50 µM, heated at 95^o^C and allowed to anneal at room temperature (1h).

Hairpin DNA (34/21) is formed by hybridization of

34bp-up : CGCGCCCAGCTTTCCCAGCTAATAAACTAAAAAC

21bp-down : GTTTTTAGTTTATTGGGCGCG

18bp DNA is formed by hybridization of

18bp-up : GTTATCCGAGCGTGAGAC

18bp-down : GTCTCACGCTCGGATAAC

## SI Table 1: Cryo-EM data collection and refinement statistics related to Figures 1 and 2.

| **Data collection and processing** | Ku-IP6  PDB: 7ZT6  EMDB: 14955 | Ku-DNA-IP6  PDB: 7ZVT  EMDB: 14986 |
| --- | --- | --- |
| Detector | Gatan K3 | Gatan K3 |
| Magnification | 130k | 130 K |
| Energy filter slit width (eV) | 20 | 20 |
| Voltage (kV) | 300 | 300 |
| Flux on detector (e/pix/sec) | 15.34 | 13.69 |
| Electron exposure on sample (e–/Å^2^) | 47.27 | 44.39 |
| Target defocus range (μm) | (-2.7) – (-0.9) | (-2.4) – (-0.8 ) |
| Calibrated pixel size (Å) | 0.652 | 0.65 |
| Symmetry imposed | C1 | C1 |
| Extraction box size (pixels) | 300 | 380 |
| Initial particle images (no.) | 485147 | 2143882 |
| Final particle images (no.) | 93175 | 276013 |
| **Refinement** |  |  |
| Map resolution at FSC=0.143 (Å)* | 3.50 | 2.74 |
| Model composition |  |  |
| Non-hydrogen atoms | 8073 | 8842 |
| Protein residues | 1031 | 1022 |
| Nucleotides  Ligands | 0  1 (IHP) | 30  1 (IHP) |
| B factor (Å^2^) |  |  |
| Protein | 170.16 | 81.29 |
| DNA  Ligands | N/A  20.00 | 121.32  151.74 |
| R.m.s deviations |  |  |
| Bond lengths (Å) | 0.005 | 0.003 |
| Bond angles (°) | 0.669 | 0.594 |
| Validation |  |  |
| Molprobity score | 1.91 | 1.64 |
| Clashscore | 13.56 | 8.12 |
| Poor rotamers (%)  Ramachandran plot  Favored (%)  Allowed (%)  Disallowed (%) | 0.70  96.1  3.90  0.00 | 1.52  97.73  2.27  0.00 |

**SI Table 2 Data collection and refinement statistics for the crystal structure of Ku70-Ku80-DNA-IP6**

| **Data collection** |  |
| --- | --- |
| Unit cell a = b, c (Å), α = β, γ (°) | 125.2, 319.7, 90°, 120° |
| Wavelength | 0.9801 |
| Space group | *P* 3_2_ 2 1 |
| Resolution range | 48.3 - 3.7 (3.79 -3.7) |
| Estimated resolution limit (Å)* | 6.2, 6.2, 3.0 |
| Total reflections | 638 939 ( 42 229) |
| Unique reflections | 31 855 (2327) |
| Unique reflections* | 12 893 (228) |
| Multiplicity | 20.1 (18.1) |
| Completeness (%) | 99.7 (98.4) |
| Completeness (%)* | 38.6 (8.8) |
| Mean *I*/sigma(*I*) | 4.2 (0.0) |
| Mean *I*/sigma(*I*)* | 10.3 (1.4) |
| Wilson B-factor | 119.52 |
| *R_merge_** | 0.220 (2.382) |
| *R_meas_** | 0.227 (2.533) |
| *R_pim_** | 0.052 (0.605) |
| CC_1/2_ | 0.996 ( 0.047) |
| CC_1/2_* | 0.997 (0.248) |
| **Refinement** |  |
| Resolution range | 48.3 - 3.7 (3.79 -3.7) |
| Reflections used in refinement | 12 771 (315) |
| Reflections used for R-free | 655 (13) |
| *R_work_* | 0.2182 (0.2924) |
| *R_free_* | 0.2729 (0.3043) |
| CC(work) | 0.921 (0.730) |
| CC(free) | 0.873 (0.604) |
| Number of non-hydrogen atoms | 8933 |
| Macromolecules | 8897 |
| Ligands | 36 |
| Protein residues | 1037 |
| RMSd(bonds) | 0.002 |
| RMSd(angles) | 0.44 |
| Ramachandran favored (%) | 95.05 |
| Ramachandran allowed (%) | 4.27 |
| Ramachandran outliers (%) | 0.68 |
| Rotamer outliers (%) | 2.78 |
| Clashscore | 4.23 |
| Average B-factor | 209.23 |
| Macromolecules | 197.82 |
| Ligands | 282.87 |

Statistics for the highest-resolution shell are shown in parentheses. Dataset from single crystal used per structure. *Values calculated after truncation by STARANISO. Estimated resolution limits along the three crystallographic directions 0.894a* - 0.447b*, b*, c*.

**SI Table 3 of IP6 contacts with Ku**

**Hydrogen Bonds** (blue line in Fig. 2C)

| **Ku70- Y416** |  | **Distance**  **with H** | **Distance without H** | **Donor angle** |
| --- | --- | --- | --- | --- |
| **7ZT6** | cryoEM Ku-IP6 | 3.20 | 4.01 | 141.19 |
| **7ZVT** | cryoEM Ku-DNA-IP6 | 3.15 | 4.07 | 167.26 |
| **7Z6O** | Xray Ku-DNA-IP6 | 3.20 | 3.63 | 109.76 |

**Salt Bridges** (yellow broken line in Fig. 2C)

| **7ZT6** | **Chain** | **Residue** | **Distance** | **Ligand group** | **7ZVT** | **Chain** | **Residue** | **Distance** | **Ligand group** |
| --- | --- | --- | --- | --- | --- | --- | --- | --- | --- |
|  | **Ku70** | K357 | 3.27 | P6 |  | **Ku70** | K357 | 3.44 | P1 |
|  |  |  | 4.01 | P5 |  |  |  | 4.81 | P5 |
|  |  |  |  |  |  |  |  | 3.29 | P6 |
|  |  | H359 | 5.01 | P5 |  |  | H359 | 4.27 | P5 |
|  |  | K443 | 4.74 | P5 |  |  | K443 | 3.82 | P3 |
|  |  |  | 4.92 | P3 |  |  |  |  |  |
|  |  |  |  |  |  |  | K445 | 5.07 | P3 |
|  |  |  |  |  |  |  |  | 3.67 | P4 |
|  |  |  |  |  |  |  |  |  |  |
|  | **Ku80** | K363 | 3.91 | P5 |  | **Ku80** | K363 | 4.85 | P4 |
|  |  |  |  |  |  |  |  | 3.86 | P5 |
|  |  | H411 | 5.39 | P5 |  |  | K411 | 4.33 | P5 |
|  |  | K413 | 4.20 | P6 |  |  | K413 | 5.40 | P4 |
|  |  |  | 5.14 | P5 |  |  |  | 3.32 | P5 |
|  |  |  |  |  |  |  |  | 3.42 | P6 |
|  |  | K481 | 3.96 | P1 |  |  | K481 | 3.68 | P3 |
|  |  |  | 5.45 | P2 |  |  |  | 3.81 | P1 |
|  |  |  | 4.92 | P3 |  |  |  | 4.74 | P2 |

| **7Z6O** | **Chain** | **Residue** | **Distance** | **Ligand group** |
| --- | --- | --- | --- | --- |
|  | **Ku70** | K357 | 4.44 | P6 |
|  |  |  | 3.41 | P1 |
|  |  |  |  |  |
|  |  | H359 | 4.66 | P5 |
|  |  | K443 | 4.89 | P3 |
|  |  | K445 | 5.45 | P4 |
|  |  |  |  |  |
|  |  |  |  |  |
|  |  |  |  |  |
|  | **Ku80** | K363 | 4.27 | P5 |
|  |  |  |  |  |
|  |  | H411 | 5.01 | P5 |
|  |  | K413 | 5.40 | P5 |
|  |  |  | 3.68 | P6 |
|  |  |  |  |  |
|  |  | K481 | 4.41 | P1 |
|  |  |  | 4.29 | P3 |

## Mass spectrometry analysis of Ku used for cryo-EM experiments. The standard of IP6 (Phytic acid sodium salt hydrate (P8810-10G, lot# BCCC6842, Sigma Aldrich UK) was made up to 10 mM and diluted 1:100 to 100 μM in ultra-pure water. The sample was analysed directly by LCMS in both acid and base modifier and stored at -20 °C when not in use. The Ku sample supplied at a final concentration of 26 μM in 20 mM Tris pH 8.0, 150 mM NaCl, 5% glycerol, 5mM DTT buffer was diluted 1:2 in water and analysed directly by UPLC-HRMS and stored at -20^O^C when not in use. The analytical system used contains a Waters Xevo Q-Tof (YBA042) mass spectrometer in ESI- ion mode scanning 150 – 850amu with leucine enkephalin as the lock mass. A Waters Acquity binary solvent manager (L09UPB913M) with column and sample manager (L09UPA552M) was used as the inlet. The chromatographic conditions were as follows:

Mobile phase A: 0.1% HCOOH/Ammonium hydroxide (aq) in Water;

Mobile phase B: 0.1% HCOOH/Ammonium hydroxide in MeCN; Conditions: 95% to 5% A over 4 minutes, 1 min equilibration; Column: 1.7 µm, CSH, C18, 2.1 x 50 mm, 40° C; Injection volume: 1 µL

## Bioinformatic analyses. Multiple sequence alignments for Ku70 and Ku80 used for evolutionary conservation mapping (SI Figure 7) were built using HHblits (1) against the uniclust30_2018_08 database with default parameters apart from a minimum coverage of 50 % with the query sequence, followed by HHfilter (1) to restrict the alignments to only homologous sequences that have at least 45 % sequence identity with the query sequence. This was done to avoid including spurious non-orthologous sequences and was still sufficient to observe contrast when mapping evolutionary conservation. These multiple sequence alignments contain only metazoan sequences, more specifically mammals, reptiles, amphibians and fish sequences. The Rate4Site algorithm (2) (used in the Consurf server (3)) was then used to derive evolutionary rates for each position on the surface of Ku70 and Ku80.

Additional sequences represented in SI figure 7 were specifically retrieved from the Uniprot database (4) for plant, yeast Ku70-Ku80 heterodimers and bacterial Ku homodimers. They were aligned to human Ku70 and Ku80 sequences by first building profiles for each sequence using HHblits against the uniclust30_2018_08 database with default parameters apart from a minimum coverage of 50 % with the query sequence, then using profile-profile alignments with HHalign (1).

## **Calorimetry**. Interactions between Ku_FL_ and inositol phosphates (IPs) (IP6 and IP6 analogues) were determined by Isothermal Titration Calorimetry (ITC) using a VP-ITC calorimeter (Malvern). Prior to measurements, all solutions were degassed under vacuum. The reaction cell of the ITC (volume 1.8 mL) was loaded with Ku heterodimers at 4 µM concentration. Proteins were dialyzed against buffer 20 mM Tris, pH 8.0, 150 mM NaCl, and 5 mM β-mercaptoethanol. The syringe (290 µL) was filled with IPs at 40 µM. The Ku heterodimer present in the cell was titrated by automatic injections of 6-10 μL of the different ligands. All binding experiments were performed at 25 °C. Enthalpy ΔH (in kcal.mol^-1^), stoichiometry of the reaction N, and association constant Ka (in M^-1^) were obtained by nonlinear least-squares fitting of the experimental data using the single set of independent binding sites model of the Origin software provided with the instrument. The free energy of binding (ΔG) and the entropy (ΔS) were determined using the classical thermodynamic formula, ΔG = - RT ln(Ka) and ΔG = ΔH - TΔS. Control experiments were performed with IPs molecules injected into the buffer to evaluate the heat of the dilution.

## switchSENSE: A DRX 2400 instrument and a multi-purpose 48bp chip were used for switchSENSE measurements (both Dynamic Biosensors GmbH; Planegg, Germany). The sample and running buffer was in 10 mM Tris HCl pH 7.4, 140 mM NaCl, 50 µM EDTA, 0.05 % Tween-20, 50µM EDTA. Kinetics experiments of Ku interaction with DNA in presence or absence of IP6 were performed on a DRX2 instrument. Complementary DNA to the strand immobilized on the chip was first hybridized on the measurement electrodes. The association and dissociation between Ku and DNA, with or without IP6 (3 µM) at Ku concentration (0-15-30-60-120-240 nM were measured in a flow rate of 200 µL/min. Kinetics experiments of Ku interaction with DNA and XLF with or without DNA were performed on a Helix instrument. The sample and running buffer was in 10 mM Tris HCl pH 7.4, 140 mM NaCl, 50 µM EDTA, 0.05 % Tween-20 with or without IP6 (20 µM). The flow rate of all conditions are at 500 µL/min.

A complementary DNA is hybridized onto an anchor DNA on the measurement chip in order to obtain a 96 base paired DNA with nick. Ku_FL_ is then captured under saturation condition (SI figure 13a and 13c). Association and dissociation between XLF and Ku-DNA is then monitored using 1, 2 or 8 µM of XLF, with or without IP6 in working buffer (SI figure 13c and d) . The fit is realised using biphasic model from BDS software.

## nano-Differential Scanning Fluorimetry. nanoDSF measurements were performed on a Tycho NT.6 device (NanoTemper) with Ku_FL_ dialyzed against buffer 20 mM Tris HCl pH 8, 150 mM NaCl, 5 mM β-mercaptoethanol. We used 9 µL of Ku_FL_ at 5 µM in absence or presence of DNA at 7.5 µM to obtain a (1:1.5) molar ratio. Measurements were performed in presence of IP6 concentrations ranging from 0 to 10 µM. Stocks solutions of IP6 and analogues are prepared so that 1 µL of IPs solutions are used in the 10 µL capillary for all concentrations tested. Each point corresponds to an average of three measurements. The temperature of transition that corresponds to the change of environment of the tryptophan is deduced from the first inflexion point of the 330 nm/350 nm ratio versus temperature.

## Fluorescent labelling of Ku. 2 µM Ku-SNAP tag protein and 4 µM SNAP-Surface Alexa Fluor 647 (New England Biolabs, NEB) were mixed and incubated at 4 ^o^C for 3 h. The mixture was washed three times using the wash buffer (20 mM Tris, 150 mM KCl, 1 mM DTT, 1 mM EDTA adjusted to pH 7.4) using amicon filter (MWCO 30K, Millipore) at ×7000 g to filter out free Alexa Fluor 647. Glycerol was added to the purified labelled protein to maintain 10 % final concentration of glycerol. The labelled protein was determined to have 50 % labelling efficiency, spectrophotometrically. The labelled protein was aliquoted and stored at -80 ^o^C.

## DNA construct preparation for single-molecule colocalization. HPLC purified oligonucleotides were obtained from Integrated DNA Technology (IDT, Coraville, IA). Oligonucleotide 1 (5’ CAT ACG ATA CAT AC/iCy3/G AGG) and oligonucleotide 2 (5’ CCT CGT ATG TAT CGT ATG /3Bio/) were annealed to prepare 10 µM of 18bpDNA construct. The DNA construct was stored at -20 ^o^C.

## Flow channel preparation for single-molecule colocalization experiment. Coverslip and drilled glass slides were functionalized using the protocol reported previously with minor modifications (5). Briefly, coverslip (Fischerfinest, size 24×50 mm) and glass slide (Fisherfinest, size 25×75×1 mm) were cleaned with 4 M KOH (Cat. 06005-1KG, Honeywell) for 30 min and washed with distilled water and methanol. (100:2:5) mixture of methanol (LC168104, Labchem), glacial acetic acid (BP1185, Thermo Fisher Scientific), and 3-(2-Aminoethylamino) propyltrimethoxysilane (Cat. A0774, TCI) was used to functionalize the glass surface with primary amine group. Glass slides and coverslips were washed, dried, and incubated with mPEG-SVA-5000 (Laysan Bio) and a mixture of mPEG-SVA-5000 and Biotin-PEG-SVA-5000 (Laysan Bio), respectively overnight in the dark. The coverslip and glass slides were washed with distilled water, dried and stored at −20 ^o^C. A microfluidic flow channel was prepared by sandwiching double-sided tapes between the drilled pegylated glass slide and biotin functionalized coverslip. Epoxy was applied at the open ends between the coverslip and glass slide of the flow channel to seal the ends. 10 µL of neutravidin solution (Invitrogen, Life Technologies Corporation, Eugene, OR) was injected from the drilled holes of the glass slide and incubated for 10 min. Excess neutravidin solution was washed with 50 µL of BSA (30 mg/mL, Gemini Bio-products) and incubated for 10 min to block the surface.

**Western-blots and antibodies.** Cell pellets were washed in PBS and resuspended in HNET buffer (50 mM Hepes-KOH, pH 7.5, 450 mM NaCl, 1 mM EDTA, 1 % Triton X-100) supplemented with Halt protease inhibitor cocktail (ThermoFisher Scientific). Cells were lysed by three freeze/thaw cycles in liquid nitrogen and 37 ˚C water bath. Lysates were cleared by centrifugation and protein concentrations were determined using the Bradford assay (Bio-Rad, Hercules, CA). Equal amounts of proteins were mixed with concentrated loading sample buffer to 1X final concentration (50 mM Tris.HCl pH 6.8, 10 % glycerol, 1 % SDS, 300 mM 2-mercaptoethanol, 0.01 % bromophenol blue), heat-denatured, separated by SDS-PAGE on Miniprotean TGX stain-free 4-15 % gradient gels (Bio-Rad, Hercules, CA) and blotted onto Immobilon-P polyvinylidene difluoride membranes (Merck-Millipore, Ireland). Membranes were blocked for 1 h in 5 % dry milk in PBS-T (phosphate-buffered saline (PBS), 0.1 % Tween-20 (Sigma-Aldrich, St. Louis, MO) and incubated for 1 h with primary antibody diluted in PBS containing 0.1 % Tween-20 and 1 % bovine serum albumin (fraction V, Sigma-Aldrich St. Louis, MO). After three washes with PBS-T, membranes were incubated for 30 min with secondary antibodies in PBS-T. Membranes were washed five times with PBS-T and immunoblots were visualized by enhanced chemiluminescence with Plus-ECL reactants (PerkinElmer, Waltham, MA). When necessary, successive immunoblotting reactions were performed on the same membrane following stripping with Restore PLUS Western Blot Stripping Buffer (Pierce, Rockford, IL). Primary antibodies used: mouse monoclonal antibodies anti-beta-Actin (clone AC-15, Ambion), anti-Ku80 (clone 111), Ku70 (clone N3H10). Peroxidase-conjugated goat anti-mouse secondary antibodies were from Jackson Immunoresearch Laboratories.

**Plasmids and DNA manipulations.**

**Ku80 expression vectors.** The untagged shRNA-resistant Ku80 lentiviral expression vector was obtained by PCR amplification using the primers Kpn2-Ku80-F and pLV-R and the previously described pLV3-HA-Ku80-shR vector (6) as a template. The resulting fragment was inserted into the Kpn2I and MluI restriction sites of the same pLV3 vector backbone. The Ku80-K481E mutant expression vector was obtained similarly following an additional step of overlap extension PCR mutagenesis with Ku80-K481E-F and Ku80-K481E-R as mutated inner primers.

**Ku70 expression vectors.** A FLAG-tagged shRNA-resistant Ku70 lentiviral expression vector was derived from the previously described pLV3-FLAG-Ku70 vector (6) by overlap extension PCR mutagenesis using Kpn2-FLAG-F and Mlu-Ku70-R as outer primers and Ku70-shR-F and Ku70-shR-R as inner primers that introduce ten silent point mutations in the shRNA target sequence of Ku70 (codons Q52-T58). The resulting fragment was subcloned into the Kpn2I and MluI restriction sites of the pLV3 vector backbone. The Ku70-K357E mutant expression vector was obtained similarly following an additional step of overlap extension PCR mutagenesis with Ku70-K357E-F and Ku70-K357E-R as mutated inner primers.

The GFP-tagged versions of FLAG-Ku70-shR-WT/K357E expressing vectors were obtained by PCR amplification of the EGFP coding sequence from the pEGFP-C1 plasmid (Clontech) using the Pme-GFP-F and Pme-GFP-R primers. The PCR fragment was then inserted at the PmeI restriction site of pLV3-FLAG-Ku70-shR-WT/K357E vectors using the Hot-Fusion strategy (7) to generate the pLV3-EGFP-FLAG-Ku70-shR-WT/K357E lentiviral vectors.

**Generation of U2OS-shKu80 cells.** The pLV-tTR-KRAB and pLVTHM2-shKu80 lentiviral vectors encoding the tTR-KRAB transcriptional repressor and allowing the conditional expression of a shRNA against Ku80, respectively, have already been described (6).

**Generation of 293T-mAID-Ku70/mAID-Ku80 cells.** To knockdown Ku70 expression, the pLVTHM2-shKu70 lentiviral vector was generated by inserting between the MluI and ClaI restriction sites of pLVTHM2 (6) the pre-annealed oligonucleotides shKu70-F and shKu70-R allowing the expression of a shRNA against codons Q52-T58 of Ku70 (8,9).

Ku80 knockout was achieved using the CRISPR-Cas9 technology by inserting the pre-annealed Ku80-gE7-F and Ku80-gE7-R oligonucleotides into the BbsI restriction sites of pCAG-eCas9-GFP-U6-gRNA plasmid (a gift from Jizhong Zou, Addgene plasmid #79145; http://n2t.net/addgene:79145 ; RRID:Addgene_79145). The resulting guide RNA targets the exon-7/intron-7 boundary of the human XRCC5/Ku80 gene (codons L161-F164), leaving all Ku80 ectopic constructs resistant to cleavage by Cas9.

A lentiviral vector expressing a PuroR-T2A-mAID-Ku70-shR construct was generated by inserting first a T2A cassette (pre-annealed oligonucleotides kpn2-T2A-Mlu-F and kpn2-T2A-Mlu-R) into the Kpn2I and MluI restriction sites of the pLV3 plasmid (6). A PCR-amplified fragment encoding a Puro-resistance cDNA (PCR reaction with primers HF-Puro-F and HF-Puro-R on a synthetic DNA molecule as a template) was then added by Hot-Fusion (7) at the Kpn2I site. The cDNA of the mini auxin-inducible degron (mAID) (10) was inserted between XmaI and EcoRI restriction sites of the previous plasmid following PCR amplification using primers Xma-mAID-F and Eco-mAID-R and the pAID1.1-N plasmid (BioROIS, Japan) as a template. Finally, the resulting vector was digested with BamHI/MluI to insert a fragment encoding Ku70-shR excised from pLV3-FLAG-Ku70-shR-WT to give the pLV3-PuroR-T2A-mAID-Ku70-shR plasmid. The pLV3-HygroR-T2A-mAID-HA-Ku80-shR vector was assembled in a similar manner, except that the HA-Ku80-shR was inserted into BamHI and SpeI restriction sites following a PCR reaction with Bam-HA-F and Spe-Ku80-R primers and the pLV3-HA-Ku80-shR plasmid as a template (6). Rice TIR1 cDNA fused to a sequence encoding three copies of c-Myc tag was PCR-amplified from the pAID1.1-N plasmid (BioROIS, Japan) with Kpn2-TIR1-F and Mlu-Myc-R primers. The resulting PCR fragment was inserted into the Kpn2I and MluI restriction sites of the pLV3 lentiviral vector (6).

**Oligonucleotides (DNA linkers and PCR primers).**

Bam-HA-F caccttGGATCCATGTACCCCTACGATGTGCctgac

Eco-mAID-R ctctcGAATTCCACGCGTcctaggtGGATCCGCTTTTATACATCCTCAAATCGATTTTCCTC

gRNA-dEJ-HR-F caccGCAACGTACGGTCTgatatc

gRNA-dEJ-HR-R aaacgatatcAGACCGTACGTTGC

HF-Puro-F GCCTCGAGGTTTAAACTACGGgatcTCCGCcATGACaGAGTACAAGCCaACaGTG

HF-Puro-R CCGCATGTTAGCAGACTTCCTCTGCCCTCGGCACCtGGCTTtCtGGTCATGCACC

Kpn2-FLAG-F ctctcgTCCGGAgccgccaccATGGACTACAAGGATG

Kpn2-Ku80-F ctcttcTCCGGAATGGTGCGGTCGGGGAATAAGGC

Kpn2-Tir1-F ctctcTCCGGAgccgccaccATGACGTACTTCCCGGAGGAGGTG

kpn2-T2A-Mlu-F CCGGAGGGCAGAGGAAGTCTGCTAACATGCGGTGACGTCGAGGAGAATCCTGGACCCGGGtcactcA

kpn2-T2A-Mlu-R CGCGTgagtgaCCCGGGTCCAGGATTCTCCTCGACGTCACCGCATGTTAGCAGACTTCCTCTGCCCT

Ku70-K357E-F CGTTGGTACTGCTGgAGAAACACCATTACCTGAGGCCCTC

Ku70-K357E-R GGTGTTTCTcCAGCAGTACCAACGGCTTGAAACCCATGAG

Ku70-shR-F CTCAatccGAgGAcGAacTcACcCCTTTTGACATGAGCATCCAGTGTATCC

Ku70-shR-R GGgGTgAgtTCgTCcTCggatTGAGATTCAAACATAGCCTTGGAGG

Ku80-gE7-F caccgAGGATCTTACAAGAATTGCA

Ku80-gE7-R aaacTGCAATTCTTGTAAGATCCTc

Ku80-K481E-F CTTGTTTCCAACCACCgAAATCCCAAATCCTCGATTTCAG

Ku80-K481E-R GAGGATTTGGGATTTcGGTGGTTGGAAACAAGTCTTCAAG

Mlu-Ku70-R ctctgcACGCGTCAGTCCTGGAAGTGCTTGGTGAGGGC

Mlu-Myc-R ctctcACGCGTctATCCGTTCAAGTCTTCTTCTGAGATTAATTTTTG

Pme-GFP-F CGATCACGAGACTAGCCTCGAGGTTTAAACGCCACCATGGTGAGCAAGGGC

pme-GFP-R ggtgcggcTCCGGAgatcCCGTAgtttGGACTTGTACAGCTCGTCCATGCCG

pLV-R CCAGTCAATCTTTCACAAATTTTGTAATCCAGAGG

shKu70-F CGCGTCCCCGAGTGAAGATGAGTTGACATTCAAGAGATGTCAACTCATCTTCACTCTTTTTGGAAAT

shKu70-R CGATTTCCAAAAAGAGTGAAGATGAGTTGACATCTCTTGAATGTCAACTCATCTTCACTCGGGGA

Spe-Ku80-R cctcttACTAGTctaTATCATGTCCAATAAATCGTCCACATCACCAC

Xma-mAID-F ctcactCCCGGGTCCAAGGAGAAGAGTGCTTGTCCTAAAG

**References**

1. Remmert, M., Biegert, A., Hauser, A. and Soding, J. (2011) HHblits: lightning-fast iterative protein sequence searching by HMM-HMM alignment. *Nat Methods*, **9**, 173-175.

2. Mayrose, I., Graur, D., Ben-Tal, N. and Pupko, T. (2004) Comparison of site-specific rate-inference methods for protein sequences: empirical Bayesian methods are superior. *Mol Biol Evol*, **21**, 1781-1791.

3. Ashkenazy, H., Abadi, S., Martz, E., Chay, O., Mayrose, I., Pupko, T. and Ben-Tal, N. (2016) ConSurf 2016: an improved methodology to estimate and visualize evolutionary conservation in macromolecules. *Nucleic Acids Res*, **44**, W344-350.

4. UniProt, C. (2021) UniProt: the universal protein knowledgebase in 2021. *Nucleic Acids Res*, **49**, D480-D489.

5. Rothenberg, E. and Ha, T. (2010) Single-molecule FRET analysis of helicase functions. *Methods Mol Biol*, **587**, 29-43.

6. Nemoz, C., Ropars, V., Frit, P., Gontier, A., Drevet, P., Yu, J., Guerois, R., Pitois, A., Comte, A., Delteil, C. *et al.* (2018) XLF and APLF bind Ku80 at two remote sites to ensure DNA repair by non-homologous end joining. *Nat Struct Mol Biol*, **25**, 971-980.

7. Fu, C., Donovan, W.P., Shikapwashya-Hasser, O., Ye, X. and Cole, R.H. (2014) Hot Fusion: an efficient method to clone multiple DNA fragments as well as inverted repeats without ligase. *PLoS One*, **9**, e115318.

8. Biard, D.S. (2007) Untangling the relationships between DNA repair pathways by silencing more than 20 DNA repair genes in human stable clones. *Nucleic Acids Res*, **35**, 3535-3550.

9. Cheng, Q., Barboule, N., Frit, P., Gomez, D., Bombarde, O., Couderc, B., Ren, G.S., Salles, B. and Calsou, P. (2011) Ku counteracts mobilization of PARP1 and MRN in chromatin damaged with DNA double-strand breaks. *Nucleic Acids Res*, **39**, 9605-9619.

10. Natsume, T., Kiyomitsu, T., Saga, Y. and Kanemaki, M.T. (2016) Rapid Protein Depletion in Human Cells by Auxin-Inducible Degron Tagging with Short Homology Donors. *Cell Rep*, **15**, 210-218.
